# Targeted inhibition of *ELANE* expression using adenine base editing to treat severe congenital neutropenia

**DOI:** 10.1016/j.omtm.2025.101626

**Published:** 2025-11-05

**Authors:** Betül Findik, Benjamin Dannenmann, Franka Bernhard, Masako Monika Kaufmann, Sandra Ammann, Sergey Kandabarau, Maksim Klimiankou, Fabian Mauch, Patrick Münzer, Oliver Borst, Isabel Klefenz, Doris Steinemann, Claudia Lengerke, Cornelia Zeidler, Toni Cathomen, Karl Welte, Masoud Nasri, Julia Skokowa

**Affiliations:** 1 Department of Hematology, Oncology, Clinical Immunology and Rheumatology, University Hospital Tübingen, Tübingen, Germany; 2 Faculty of Medicine University Tübingen, Gene and RNA Therapy Center (GRTC), Tübingen, Germany; 3 Institute for Transfusion Medicine and Gene Therapy, Medical Center – University of Freiburg, Freiburg, Germany; 4 Center for Chronic Immunodeficiency (CCI), Faculty of Medicine, University of Freiburg, Freiburg, Germany; 5 Spemann Graduate School of Biology and Medicine (SGBM), University of Freiburg, Freiburg, Germany; 6 Severe Chronic Neutropenia International Registry, SCNIR, Hannover, Germany; 7 DFG Heisenberg Group Cardiovascular Thromboinflammation and Translational Thrombocardiology, University of Tübingen, Tübingen, Germany; 8 Department of Cardiology and Angiology, University Hospital Tübingen, Tübingen, Germany; 9 Department of Human Genetics, Hannover Medical School, Hannover, Germany; 10 German Cancer Consortium (DKTK), partner site Tübingen, a partnership between DKFZ and University Hospital Tübingen, Tübingen, Germany; 11 Department of Pediatric Hematology, Oncology and Bone Marrow Transplantation, Children’s Hospital, University Hospital Tübingen, Tübingen, Germany

**Keywords:** severe congenital neutropenia, adenine base editing, hemopoietic stem cells, gene therapy, bone marrow failure syndrome, neutrophils, *ELANE*, NETs, pre-leukemia, neutrophil elastase

## Abstract

Autosomal dominant mutations in *ELANE* (elastase, neutrophil expressed) cause severe congenital neutropenia (CN) and cyclic neutropenia (CyN). Inhibiting *ELANE* expression, either by CRISPR-Cas9-mediated *ELANE* knockout or promoter targeting using CRISPR-Cas9 nickase, has emerged as a promising gene therapy strategy to restore defective granulocytic differentiation of transplantable hematopoietic stem cells from CN patients. We developed an adenine base editor (ABE)-mediated approach targeting two nucleotides in the *ELANE* promoter to suppress neutrophil elastase expression, called PRECISE. Analysis of mRNA- and protein-based delivery of ABE revealed that although both platforms were effective in editing hematopoietic stem and progenitor cells from healthy donors with over 80% editing, only protein-based ABE delivery achieved over 68% editing in CN patient cells. Interestingly, 10%–19% editing in CN patients’ hematopoietic cells using ABE mRNA restored their granulocytic differentiation *in vitro*, with a marked expansion and differentiation of ABE ribonucleoprotein (RNP)-edited cells. PRECISE-edited neutrophils retained normal function, including neutrophil extracellular trap formation, oxidative burst, and phagocytosis. Genome integrity analysis showed no genomic alterations or chromosomal aberrations, and only two off-target edits confined to non-coding intronic regions. In conclusion, PRECISE represents a translationally relevant base-editing strategy for *ELANE*-associated CN and CyN that addresses *ELANE* mutation heterogeneity.

## Graphical abstract

**Figure:**
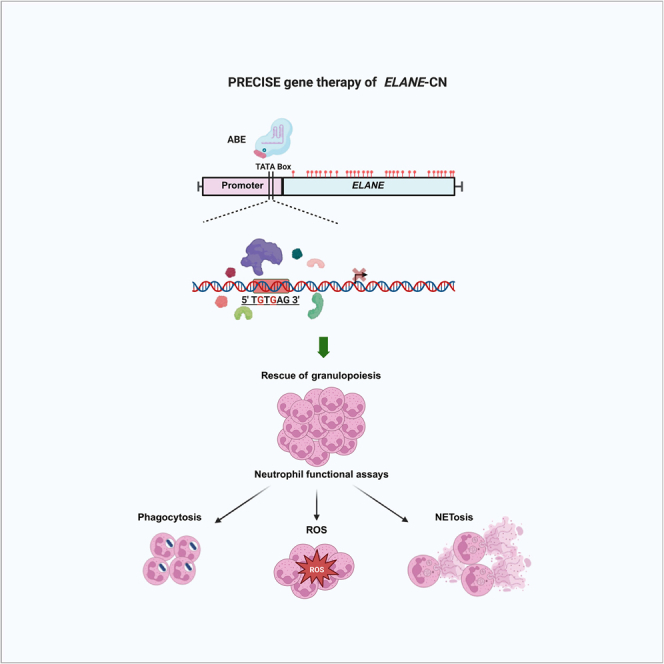

## Teaser

PRECISE, an adenine base-editing strategy for *ELANE*-CN HSPCs, converts two adenines in the *ELANE* promoter TATA box to guanines using ABE8.20-m, thereby inhibiting *ELANE* expression and restoring granulopoiesis with a favorable off-target profile and no genomic aberrations. This approach is promising for gene therapy of *ELANE*-CN.

## Introduction

Several gene-editing strategies for autosomal dominant *ELANE* mutations associated with severe congenital neutropenia (CN)^1^ or cyclic neutropenia (CyN) were reported to be suitable for potential clinical translation.^2^^,^^3^^,^^4^ CN and CyN are rare heterogeneous pre-leukemia bone marrow failure syndromes, and *ELANE* mutations are the main genetic cause of CN. More than 120 *ELANE* variants have been reported, most of which have a damaging effect on myeloid progenitor cells.^5^ We and others have reported that inhibiting *ELANE* expression in *ELANE*-CN hematopoietic stem and progenitor cells (HSPCs) using CRISPR-Cas9-mediated gene knockout could be a universal therapeutic approach for these patients, regardless of the mutation position, successfully restoring granulopoiesis *in vitro* and *in vivo*.^2^^,^^3^^,^^4^ We have also recently demonstrated that introducing small deletions within *ELANE*’s promoter TATA box using CRISPR-Cas9 nickases can be a universal, safe, and effective gene therapy approach for *ELANE*-CN.^6^ This strategy reduces *ELANE* mRNA expression levels without targeting the coding DNA sequence, thereby avoiding the generation of *de novo ELANE* mutant variants and rescuing granulopoiesis in *ELANE*-CN patients. Application of Cas9 nickases reduced the probability of unintended CRISPR-Cas9-induced double-strand breaks (DSBs), which can result in various potential genotoxic effects and detrimental changes to the HSPCs pool.^7^ However, there is still a chance of unwanted DSBs even if Cas9 nickase is used.^8^ Similarly, while CRISPR-Cas9 adenine base editors (ABEs) significantly reduce the likelihood of DSBs, they are not entirely DSB-free, as the chance of such events is close to but not zero. To enhance precision and minimize potential genotoxic effects, we sought to develop a highly precise approach by investigating the use of ABE to target the *ELANE*’s TATA box, aiming to achieve similar therapeutic benefits with an improved safety profile.

Base editors allow for precise modification of single base pairs within the genomic DNA sequence, enabling targeted therapeutic intervention. Base editors are created by fusing a nucleobase deaminase enzyme and, in some cases, a DNA glycosylase inhibitor, to the catalytically disabled CRISPR nuclease, allowing for the direct modification of single base pairs in DNA or RNA without introducing DSBs into the genome.^9^ ABEs convert adenine (A) to hypoxanthine, which is subsequently repaired by cellular processes to guanine (G), resulting in an A-to-G base conversion. They typically generate high product purity with no reported cases of significant A-to-non-G edits.^9^^,^^10^ Base editing has been employed to target *cis*-regulatory elements of disease-causing genes, thereby modulating their expression levels. For example, Lim et al. utilized base editing to target non-coding regions within the promoter of the human huntingtin (*HTT*) gene, implicated in Huntington’s disease, and the amyloid precursor protein gene, associated with Alzheimer’s disease. By altering these regulatory elements, they aimed to control corresponding gene expression and explore potential therapeutic strategies for neurodegenerative diseases.^11^ Zeng et al. and Liao et al. also demonstrated that a single therapeutic base edit within the *BCL11A* enhancer effectively suppressed sickle hemoglobin production and ameliorated globin chain imbalance in erythroid cells derived from patients with sickle cell disease and HSPCs from β-thalassemia patients.^12^^,^^13^ Huang et al. demonstrated the efficacy of ABE in plants by targeting the TATA box of the *LOB1* gene promoter in grapefruit (*Citrus paradisi*) and sweet orange (*Citrus sinensis*).^14^ This precise modification converted the TATA box to a CACA motif, successfully conferring resistance to citrus canker.

Here, we report a novel gene therapy approach to inhibit *ELANE* mRNA expression by converting two adenine nucleotides to guanine nucleotides in the *ELANE* promoter’s TATA box using ABE8.20-m. This strategy minimizes DSBs, offering a precise and universal solution for reducing *ELANE* expression and restoring granulopoiesis in *ELANE*-CN patients. We also demonstrated a favorable safety profile of ABE editing of *ELANE* in primary HSPCs, performing CAST-seq, optical genome mapping, RNA sequencing (RNA-seq), and rhAmpSeq. These findings demonstrate the potential of ABE as a curative therapy for *ELANE*-CN patients.

## Results

### Inhibition of *ELANE* mRNA expression by ABEs

**Figure 1:**
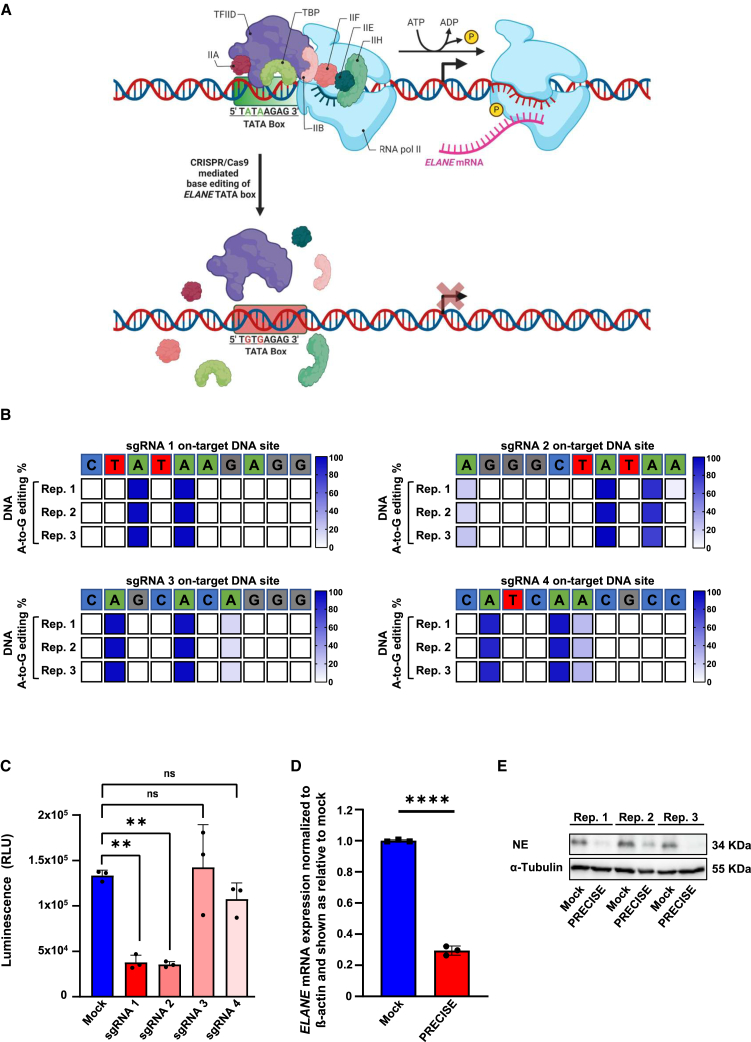
Establishment of a precise double-strand break-free strategy for reducing *ELANE* mRNA expression through adenine base editing of the *ELANE* gene promoter (A) Scheme of adenine base-editing strategy, targeting the *ELANE* promoter’s TATA box, termed PRECISE. The image illustrates the key steps in transcription initiation in eukaryotes, focusing on the promoter region containing the TATA box. The TATA binding protein (TBP), a subunit of TFIID, is the first protein to bind DNA, initiating the formation of the RNA polymerase II (RNA Pol II) preinitiation complex (PIC). This binding facilitates the recruitment of the general transcription factors and RNA Pol II to the promoter. The sequential binding of TFIID, TFIIB, TFIIA, RNA Pol II, TFIIF, TFIIE, and TFIIH leads to the formation of the PIC. TFIIH uses ATP hydrolysis to unwind the DNA and initiate transcription. Adenine base editing of the TATA box prevents TBP from binding, thereby disrupting PIC formation. This figure was adapted and modified from “Regulation of Transcription in Eukaryotes: A Molecular Approach” by GM Cooper^15^ and created using BioRender.com. (B) Heatmap of on-target DNA base editing efficiencies of ABE8.20-m mRNA and *ELANE* promoter targeting gRNAs in NE-HiBiT-tagged THP-1 reporter cell line. Bases shown are within the editing window of the on-target site. The percentage of on-target base editing conversion A-to-G is reported using BEAT algorithms from Sanger sequencing data obtained 5 days post electroporation. Rep 1 to 3 represents the data from three independent experiments. (C) Neutrophil elastase (NE) expression levels in NE-HiBiT-tagged THP-1 reporter cells were measured 120 h post electroporation with several sgRNAs targeting the *ELANE* promoter region upstream of the transcription start site. Luminescence signal was quantified using the GloMax system. Data are presented as relative luminescence units (RLU) as mean ± standard deviation (SD) from three independent experiments. One-way ANOVA with Dunnett’s multiple comparison test was used to assess statistical significance. ∗∗*p* < 0.01, ns: not significant. (D) *ELANE* mRNA expression in NE-HiBiT-tagged THP-1 reporter cell line was measured by quantitative reverse-transcription PCR 14 days post electroporation of the mock or PRECISE-edited cells. *ELANE* mRNA expression was normalized to β-actin and is shown relative to the mock group. Data represent means ± SD from three independent experiments. Unpaired Student’s *t* test was applied for group comparison, ∗∗∗∗*p* < 0.0001. (E) Representative western blot images of NE and α-tubulin of protein lysates 14 days post electroporation of mock and PRECISE-edited THP-1 cells from three independent experiments.

We have previously demonstrated that the introduction of a small deletion within the *ELANE* TATA box inhibits *ELANE*’s mRNA expression.^6^ We now investigated whether ABE of the *ELANE* TATA box could inhibit *ELANE* transcription in a more precise, DNA DSB-free manner (Figure 1A). First, we screened the base conversion effect of four single-guide RNAs (sgRNAs), selected based on their position on the *ELANE* core promoter, as described previously^6^ by electroporating the THP-1 *ELANE*-HiBiT reporter cell line—constitutively expressing high levels of HiBiT-tagged neutrophil elastase (NE)—with each of four sgRNAs and ABE mRNA (ABE8.20-m). The THP-1 *ELANE*-HiBiT reporter cell line generation and sequences of the corresponding sgRNAs and repair template have been described elsewhere.^6^ The on-target editing efficiency was assessed by analysis of Sanger sequence traces 5 days post electroporation using Base Editing Analysis Tool (BEAT) algorithm^16^ (Figures 1B and S1A–S1D). All selected sgRNAs efficiently induced A-to-G conversions within their respective base editing windows. Both sgRNAs 1 and 2, which targeted the TATA box, exhibited similar editing and functional efficiencies. sgRNA 1 converted two adenines to guanines (A2: >90% and A1: >90%), and sgRNA 2 converted three adenines to guanines (A3: >15%, A2: >90%, A1: >90%). sgRNAs 3 and 4, targeting the ELANE promoter upstream of the TATA box, were used as controls. The modification of the TATA box using mRNA ABE in the THP-1 reporter cell line led to markedly reduced *ELANE* expression levels 5 days post electroporation, as assessed by measuring the luminescence levels of endogenously tagged NE protein (Figure 1C). Due to its uniform effect, sgRNA 1 was selected for further development. qPCR and western blot analyses of *ELANE* mRNA and protein expression levels in the reporter THP-1 cell line also confirmed marked reduction of *ELANE* and NE levels in the ABE-edited group using sgRNA 1 (Figures 1D and 1E).

In summary, ABE can be used to inhibit *ELANE* expression by TATA > TGTG conversion in the TATA box of the *ELANE* promoter. We have termed the proposed gene-editing strategy PRECISE.

### Granulocytic differentiation of healthy donor HSPCs remained unaffected by PRECISE editing

**Figure 2:**
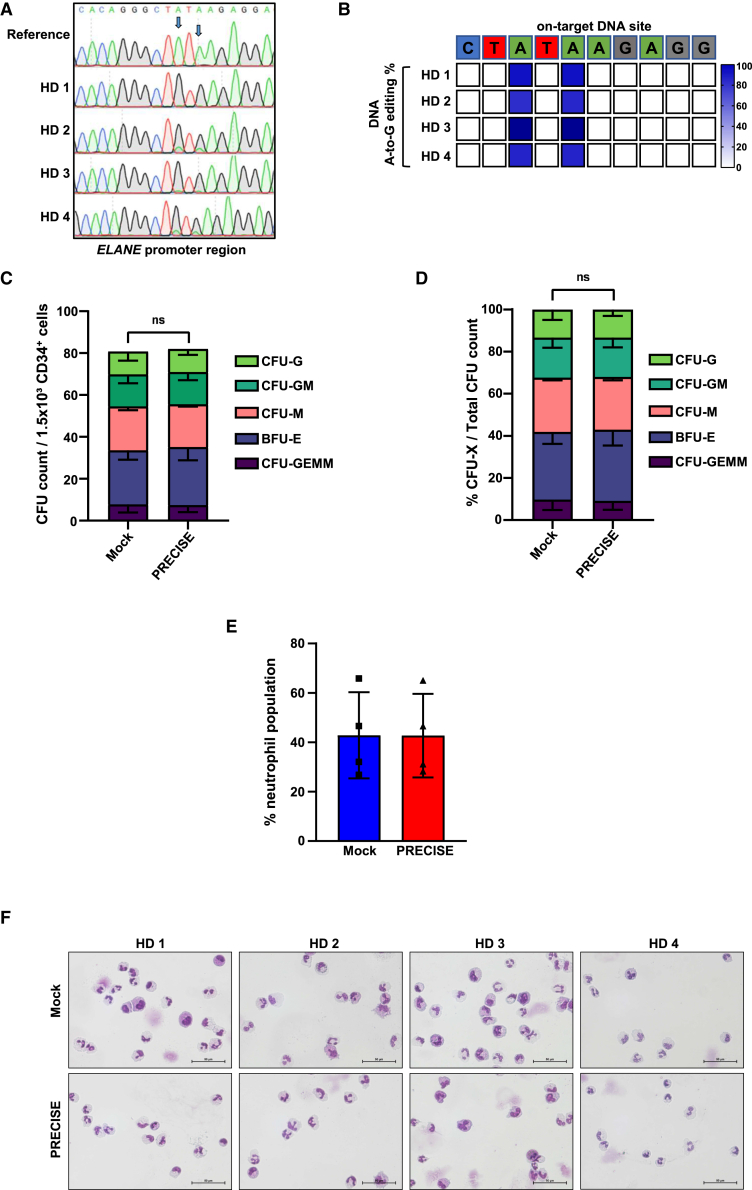
PRECISE did not affect granulocytic differentiation of healthy donor HSPCs (A) Representative Sanger sequencing traces of DNA isolated from four healthy donor HSPCs 5 days post electroporation with PRECISE. Arrows indicate the target sites for base editing within the DNA sequence. (B) Heatmap of on-target DNA base editing efficiencies of PRECISE in HD 1–4 patients’ HSPCs. Bases shown are within the editing window of the on-target site. The percentage of on-target base editing conversion A-to-G is reported using BEAT algorithms from Sanger sequencing data obtained 5 days post electroporation. HDs 1–4 represent the data from four independent experiments. (C and D) CFU assay of CD34^+^ HSPCs isolated from four healthy donors (HD 1–4) after mock or PRECISE electroporation. Data are represented as mean ± SD of CFU counts (C) or percentages (D) from HD 1–4 in duplicates, including group comparison, ns: not significant. (E) Flow cytometry analysis of CD45^+^CD16^+^CD66b^+^ neutrophil population after 14 days of LCD of mock vs. base-edited CD34^+^ HSPCs of HD 1–4. Data represent means ± SD from four independent experiments, each in duplicates. (F) Representative images of May-Grunwald-Giemsa-stained cytospins of neutrophils after 14 days of LCD for HD 1–4 CD34^+^ HSPCs. Scale bars represent 50 μM.

We next assessed the impact of PRECISE editing on granulocytic differentiation of CD34^+^ HSPCs from four healthy donors (HDs) *in vitro*. PRECISE editing efficiency 5 days post electroporation was between 83% and 97%, as assessed by BEAT algorithm^16^ using Sanger sequencing data (Figures 2A, 2B, and S2). Colony-forming unit (CFU) assay showed no significant difference in colony distributions, neither in absolute count nor in percentage, between the PRECISE and mock groups, which serve as positive control for granulocytic differentiation (Figures 2C and 2D). Analysis of neutrophil-specific surface markers by FACS at day 14 of liquid culture differentiation (LCD) toward neutrophils and morphological examination of cells on cytospin slides also showed comparable results between the PRECISE and mock-electroporated groups (Figures 2E and 2F).

### PRECISE editing preserved the function of *in vitro*-generated neutrophils

**Figure 3:**
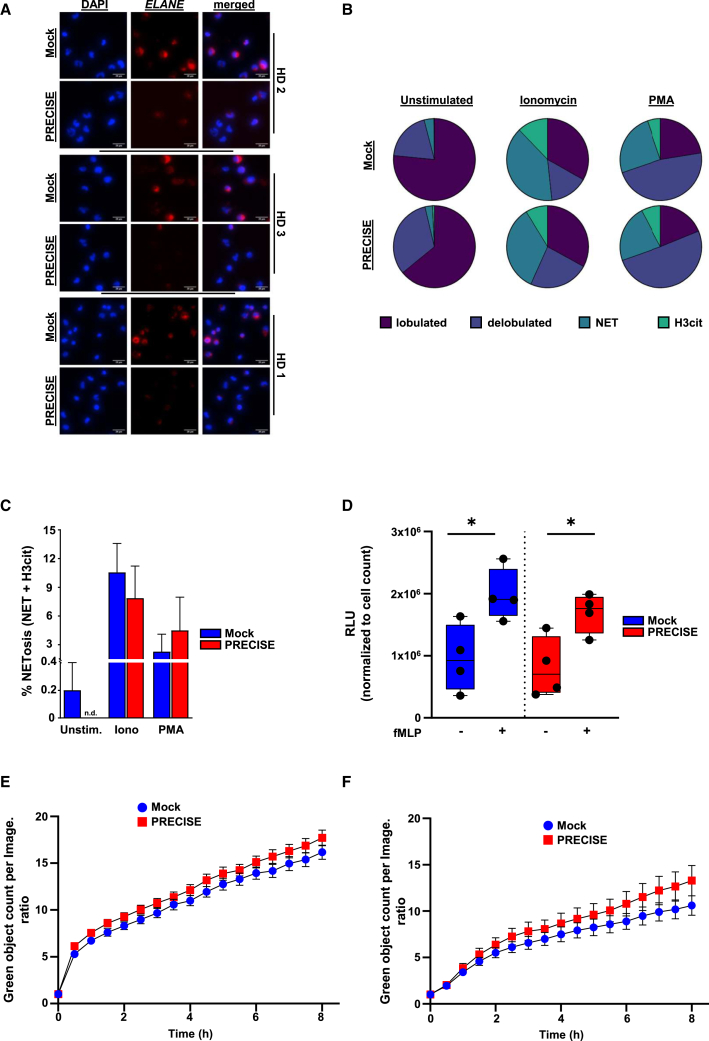
PRECISE did not affect NETosis, ROS generation, or phagocytosis capabilities of *in vitro*-generated neutrophils (A) Representative fluorescence images of NE expression in mock- and PRECISE-treated *in vitro*-generated neutrophils from three healthy donors (HD 1–3). Scale bars represent 20 μm. (B) Average occurrence of depicted cellular NETosis stages in mock- and PRECISE-treated *in vitro*-generated neutrophils, both unstimulated and stimulated with ionomycin (4 μM) or PMA (100 nM) for 4 h. (C) Arithmetic means ± SEM (*n* = 3; right) of NET formation in mock- and PRECISE-treated *in vitro*-generated neutrophils in the absence (unstimulated) or presence of ionomycin or PMA for 4 h. (D) Levels of hydrogen peroxide (H_2_O_2_) reactive oxygen species (ROS), measured after stimulation of *in vitro* differentiated mock- and PRECISE-derived neutrophils with formylmethionine-leucyl-phenylalanine (fMLP) for 30 min. Data represent means ± SD from four biological replicates, each in duplicates. ∗*p* < 0.05. (E and F) Phagocytosis kinetics of pHrodo Green *S. aureus* bioparticles (E) and pHrodo Green Zymosan bioparticles (F) by neutrophils derived from liquid differentiation culture on day 14 of mock and PRECISE edited HSPCs, performed and analyzed using the IncuCyte Zoom System. The Y-axis data represents the relative increase in phagocytic activity over the presented time course. It was calculated as the ratio of the Green Object Count measured at each time point (tx) normalized to the Green Object Count measured at the initial time point (*t* = 0) within the same image frame. Data represent means ± SD from three biological replicates, each in duplicates.

We further assessed the ability of the *in vitro*-generated PRECISE-edited neutrophils to perform key functions, including neutrophil extracellular trap (NET) formation, oxidative burst, and phagocytosis of bacteria and fungi, in the absence of *ELANE* expression. Immunofluorescence staining of PRECISE-edited neutrophils, used in functional assays, revealed a marked decrease in NE protein expression compared to control cells, as demonstrated by an NE-specific antibody (Figure 3A). The absence of NE did not impair their NET formation capability, as assessed by the quantification of the average occurrence of several cellular stages of NETosis in neutrophils stimulated with either ionomycin or phorbol myristate acetate (PMA), both known *in vitro* inducers of NETosis (Figures 3B and 3C). Furthermore, the ability of *in vitro*-generated neutrophils to produce reactive oxygen species (ROS) in response to stimulation with formyl-methionyl-leucyl-phenylalanine (fMLP) was also not affected upon *ELANE* inhibition (Figure 3D). Using live-cell imaging (IncuCyte ZOOM), phagocytosis of *S. aureus* (pHrodo green *S. aureus*) and yeast-derived cell wall particles (pHrodo green Zymosan) was also preserved in PRECISE-edited neutrophils (Figures 3E and 3F).

### PRECISE editing is a promising therapeutic approach for *ELANE*-CN with a favorable safety profile

**Figure 4:**
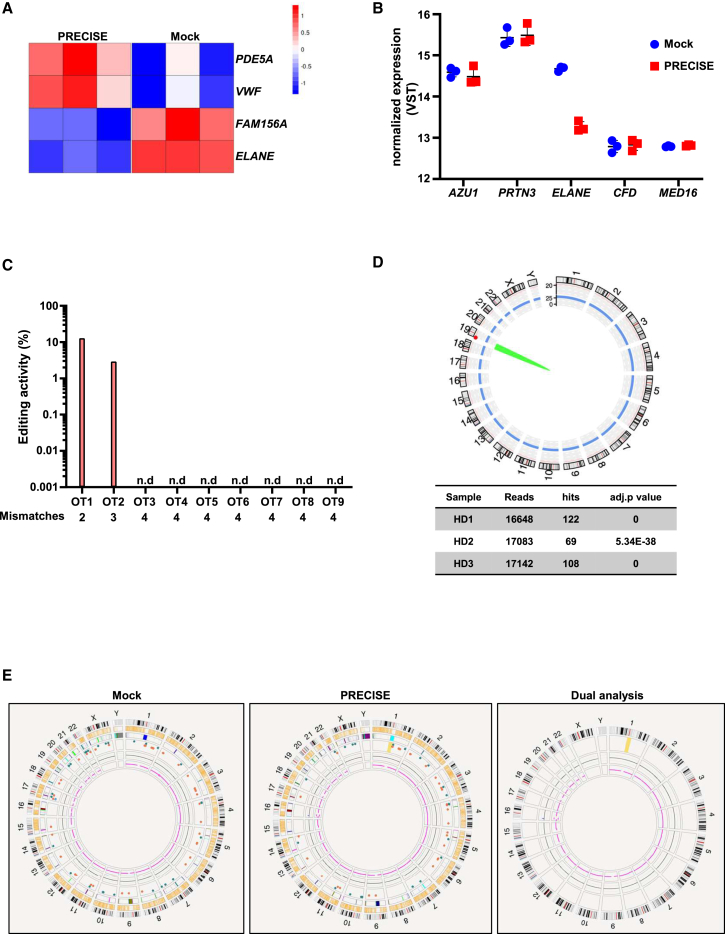
Safety profiling of PRECISE (A) Heatmap showing differentially expressed genes (DEGs) in PRECISE-edited healthy donor HSPCs (*n* = 3) 5 days post electroporation, compared to mock. The color scale represents log2 fold change values, with red indicating increased expression and blue indicating decreased expression. (B) Normalized expression levels of genes neighboring *ELANE* in PRECISE-edited healthy donor HSPCs (*n* = 3) 5 days post electroporation, compared to mock. VST, variance stabilizing transformation. (C) Potential *in silico*-predicted off-target sites (OTs) identified by rhAmpSeq in mock and PRECISE-edited healthy donor HSPCs (HD 1–3) 5 days post electroporation. NGS data were analyzed using the CRISPRESSO pipeline. Sequencing data from HD 1–3 (*n* = 3) were pooled before analysis. (D) CAST-seq for the qualitative evaluation of chromosomal rearrangements in PRECISE-edited healthy donor HSPCs (*n* = 3) 5 days post electroporation. The Circos plot visualizes on-target site aberrations (green) and off-target-mediated translocations (red; not detected). (E) Circos plots from optical genome mapping (OGM). From outer to inner track (mock and PRECISE): ideograms of the chromosomes 1–22, X, Y; hg38 gene feature; DGV GS hg38 2026 feature; SVs as colored dots, blue: duplication, orange: deletion, green: insertion; copy number counts: zero line in black = 2n, +1 = 3n, −1 = 1n; pink line: translocations (if present). Next to mock and PRECISE, the dual analysis is shown as circos plot in which data for PRECISE are calculated against mock. No structural variants are called, which are found in the PRECISE but not in the mock sample except for one mosaic CNV at chr 16p that reflects a common polymorphism and is most likely due to mitotic recombination without clinical relevance. Note: tracks for genes and DGV (database of genomic variants: http://dgv.tcag.ca/dgv/app/home) is not given in the dual analysis. All genomic coordinates are based on the human reference genome assembly GRCh38.

To further investigate the specificity of the PRECISE approach in reducing *ELANE* mRNA expression levels, we performed RNA-seq analysis of mock and PRECISE-edited CD34^+^ HSPCs from three HDs 5 days post electroporation (Figures 2A and 2B). We found only four differentially expressed genes with a fold change greater than 2 and a false discovery rate less than 0.05. mRNA expression of *PDE5A* and *VWF* was upregulated, while *FAM156A* and *ELANE* mRNA expression was downregulated (Figures 4A and S7A). *ELANE* showed a significant 6.6-fold downregulation (log2-fold change = −2.72, adjusted *p* value = 1.854e−49). No significant changes in the expression levels of the *ELANE*-neighboring genes *PRTN3*, *AZU1*, *MED16*, and *CFD* were observed (Figures 4B and S7B). To assess the potential off-target effects of the PRECISE editing, we designed a targeted amplicon sequencing (rhAmpSeq) panel, which includes nine guide RNA (gRNA)-dependent genomic sites identified by CRISPRitz^17^ as potential off-target sites for the applied gRNAs, based on their Cutting Frequency Determination scores (Table 1). rhAmpSeq libraries were generated and sequenced from genomic DNA from three HDs 5 days post nucleofection with mock or PRECISE editing (Figures 2A and 2B). Using CRISPRESSO^18^-based analysis of sequencing data, we observed detectable off-target editing at two sites. Off-target (OT) site 1 (OT1) is located on chr8:130168778-130168800 in the intronic region of the *ASAP1* gene, with two mismatches to the target gRNA. Off-target site 2 (OT-2) is located on chr6:135305934-135305956 in the intronic region of the *AHI1* gene, with three mismatches to the target gRNA (Figures 4C, S3A, and S3B). Neither of the OT sites revealed overlap with any known regulatory elements in ENCODE data portal,^19^ suggesting that these off-target modifications are unlikely to disrupt gene expression or cellular function. Also, ClinVar database^20^ stores no short variants with known clinical significance in the introns of both genes. Additionally, PRECISE did not affect the expression levels of genes located at identified OTs (Figure S7C). No off-target editing was detected at the sites with four mismatches to the target gRNA. We also evaluated the chromosomal integrity of PRECISE-edited primary CD34^+^ HSPCs of three HDs, applying CAST-seq, a sensitive method for detecting chromosomal rearrangements.^21^ While we observed few on-target aberrations, no off-target chromosomal translocations and other structural changes were identified (Figure 4D). Optical genome mapping (OGM) on PRECISE-edited primary CD34^+^ HSPCs from HD2 5 days post electroporation (Figures 2A and 2B) was performed to evaluate potential introduction of structural variations (SVs) in the genome of PRECISE-edited cells. No insertions, deletions, inversions, or other SVs were found between mock and PRECISE samples (Figures 4E and S3C).

**Table 1:** Potential *in silico*-predicted off-target sites

Off-target site	Chromosome	Position	Mismatches
OT1	chr8	130168777	2
OT2	chr6	135305933	3
OT3	chr1	103107538	4
OT4	chrX	148803588	4
OT5	chr12	93377037	4
OT6	chr4	25008773	4
OT7	chr2	241983480	4
OT8	chr12	52701183	4
OT9	chr20	4919449	4

These findings suggest that the PRECISE approach has a favorable safety profile, underscoring its potential for clinical translation.

### PRECISE editing restored granulocytic differentiation of CN patients’ HSPCs

**Figure 5:**
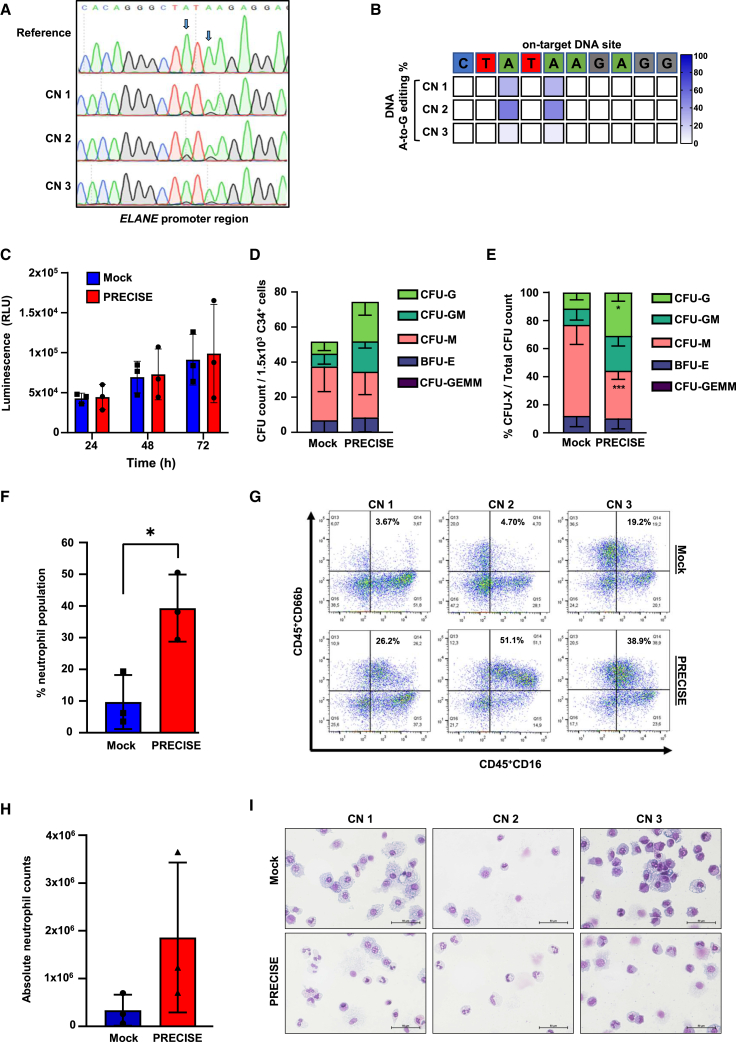
PRECISE-editing restored granulocytic differentiation of CN patients’ HSPCs *in vitro* (A) Representative Sanger sequencing traces demonstrating base editing of the *ELANE* gene promoter. The reference sequence is shown at the top, followed by traces from three independent experiments (CN1, CN2, and CN3). Arrows indicate the target sites for base editing within the DNA sequence. (B) Heatmap of on-target DNA base editing efficiencies of PRECISE in CN1–3 patients’ HSPCs 3 days post electroporation. Bases shown are within the editing window of the on-target site. The percentage of on-target base editing conversion A-to-G is reported using BEAT algorithms from three independent experiments (CN1–3). (C) RealTime-Glo MT cell viability assay was performed in edited CD34^+^ HSPCs at 24, 48, and 72 h post electroporation for mock and PRECISE. Luminescence signal (RLU) was quantified using the GloMax system. Data represent the mean ± SD from three independent experiments (CN1–3), each measured in duplicates. (D and E) CFU assay of the PRECISE-edited CD34^+^ HSPCs of three CN patients (CN1–3) compared to mock sample. CFU colony counts (D) and percentages (E) are shown. Data represent means ± SD from CN1–3 in duplicates. Group comparisons are shown, ∗*p* < 0.05, ∗∗∗*p* < 0.001. (F–H) Flow cytometry analysis of CD45^+^CD16^+^CD66b^+^ neutrophil population after 14 days of LCD of mock sample and PRECISE-edited CD34^+^ HSPCs of three CN patients (CN1–3). Data represent means ± SD from CN1–3, each in duplicates, including group comparisons, ∗*p* < 0.05. Percentage of neutrophil population (F) or absolute neutrophil counts are depicted (H). Representative flow cytometry plots are shown for each CN patient (G). (I) Representative images of May-Grunwald-Giemsa-stained cytospin preparations on day 14 of LCD of mock and PRECISE-edited HSPC-derived cells for three CN patients (CN1–3). Scale bars represent 50 μm.

To further evaluate the efficacy of PRECISE editing as a potential treatment for *ELANE*-CN, we tested it in primary bone marrow-derived CD34^+^ HSPCs from three *ELANE*-CN patients, harboring *ELANE* mutations (NP_001963.1): CN1: p.Trp156Cys, CN2: p.F199LfsX16, and CN3: p.Arg103Pro (Figure S4A). The base editing efficiency was 14%, 19%, and 11% 3 days post electroporation and reached 34%, 53%, and 9% after 14 days of neutrophilic differentiation for CN1, CN2, and CN3 patients, respectively, as assessed by the BEAT algorithm using Sanger sequencing data obtained from cells electroporated with ABE8.20-m mRNA and sgRNA 2 (Figures 5A, 5B, S4B, and S4C). The PRECISE approach did not induce any detectable cytotoxicity in *ELANE*-CN gene-edited CD34^+^ HSPCs, as assessed by RealTime-Glo MT cell viability assay at 24, 48, and 72 h post electroporation, when compared to a mock-electroporated control group (Figure 5C). Assessing granulocytic differentiation capacity of PRECISE gene-edited cells, we detected a significant increase in the percentage of granulocytic colony-forming units (CFU-G) in the PRECISE group, when compared to the mock-electroporated control group. Nonetheless, no statistically significant difference was found in the absolute count of CFU-G (Figures 5D and 5E). *In vitro* granulocytic liquid culture differentiation assay also revealed that granulopoiesis was restored on day 14 of differentiation compared to the mock-electroporated control group, as assessed based on the percentage and count of cells expressing the neutrophil-specific markers CD45^+^CD16^+^CD66b^+^ (Figures 5F–5H) as well as evaluation of cell morphology of May-Grunwald-Giemsa-stained cytospin images of mock or gene-edited cells on day 14 of culture (Figure 5I). Notably, the percentage of edited cells increased for two CN patients (CN1 and CN2) during 14 days of granulocytic differentiation culture, clearly demonstrating their differentiation advantage (Figure S4C). For patient CN3, editing remained stable, corresponding to the patient’s weak differentiation (see cell morphology on cytospin slides, Figure 5I), combined with a milder CN phenotype and a good response to granulocyte colony stimulating factor (G-CSF).

Taken together, these findings demonstrate that PRECISE is a promising gene therapy strategy for *ELANE*-CN. However, the editing efficiency of mRNA-delivered ABE8.20-m in *ELANE*-CN HSPCs did not exceed 19% 3 days post electroporation, which is in a sharp contrast to the 83%–97% editing efficiency observed in HDs.

### Ribonucleoprotein delivery of PRECISE outperforms mRNA for gene editing in *ELANE*-CN HSPCs

We observed a significantly lower base editing efficiency in CN HSPCs when ABE8.20-m mRNA was used in the electroporation, as compared to HDs (10%–19% in CN vs. 83%–97% in HDs). We hypothesized that this difference might be due to reduced ABE8.20-m mRNA translation efficiency in CN patient HSPCs compared to HDs. To test this, we first performed PRECISE editing using ABE8-20-m ribonucleoprotein (RNP) in the THP-1 *ELANE*-HiBiT reporter cell line. On-target base editing efficiency of ABE8.20-m RNP was comparable to the ABE8.20-m mRNA (Figure S5A); NE expression was also significantly reduced 72 h post electroporation (Figure S5B). Using Sanger sequencing, we were not able to detect off-target editing at the OT1 site (Figure S5C).

**Figure 6:**
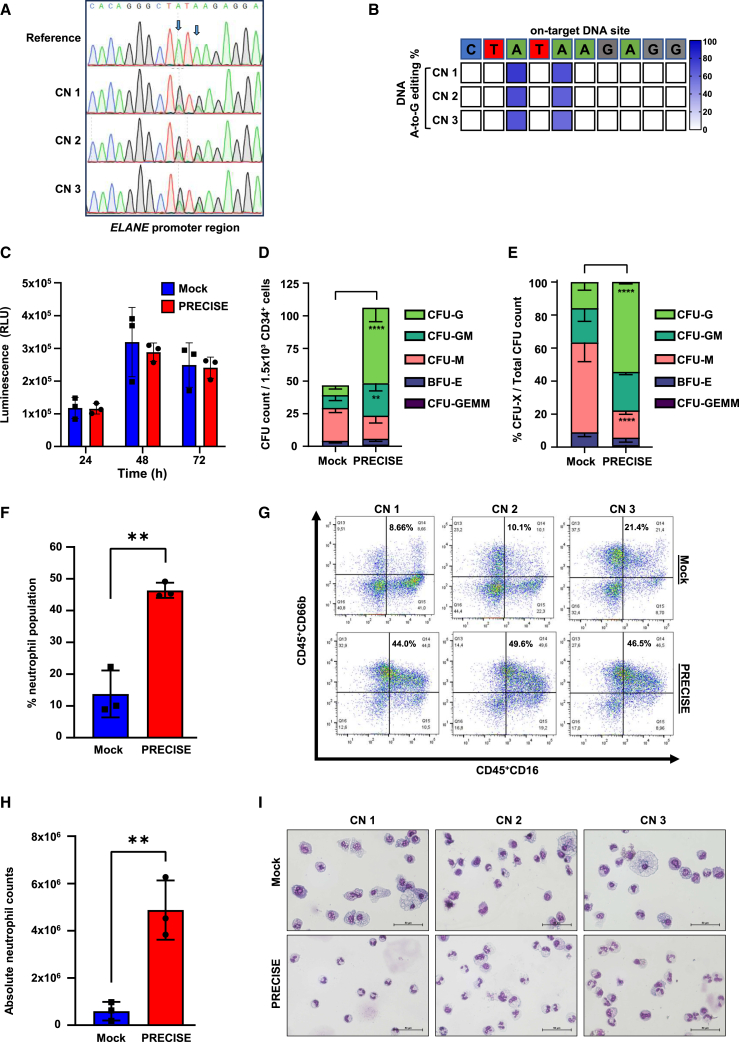
Enhanced gene editing in *ELANE*-CN HSPCs with RNP-delivered ABE compared to mRNA (A) Representative Sanger sequencing traces of base-edited *ELANE*-CN HSPCs. Data represent three independent experiments. Arrows indicate the target sites for base editing within the DNA sequence. (B) Heatmap depicting on-target DNA base editing efficiencies of PRECISE in three patients’ HSPCs (CN1–3) 3 days post electroporation. BEAT algorithm was used to report the on-target base editing conversion A-to-G in percentage from three independent experiments (CN1–3). (C) RealTime-Glo MT cell viability assay was performed at 24, 48, and 72 h post electroporation for mock sample and PRECISE (RNP form) in edited CD34^+^ HSPCs. Luminescence signal (RLU) was quantified using the GloMax system. Data represent the mean ± SD from three independent experiments (CN1–3), each measured in duplicates. (D and E) CFU assay of the PRECISE-edited CD34^+^ HSPCs of three CN patients (CN1–3) compared to mock sample. CFU colony counts (D) and percentages (E) are shown. Data represent means ± SD from three CN patients (CN1–3) measured in duplicates. Group comparisons are shown, ∗∗*p* < 0.01, ∗∗∗∗*p* < 0.0001. (F–H) Flow cytometry analysis of CD45^+^CD16^+^CD66b^+^ neutrophil population after 14 days of LCD of mock sample and PRECISE-edited CD34^+^ HSPCs of three CN patients (CN1–3). Data represent means ± SD from CN1–3, each in duplicates, including group comparisons, ∗∗*p* < 0.01. Percentage of neutrophil population (F) and absolute neutrophil counts are depicted (H). Representative flow cytometry plots are shown for each CN patient (G). (I) Representative images of May-Grunwald-Giemsa-stained cytospin preparations on day 14 of LCD of mock and PRECISE-edited HSPC-derived cells for three CN patients (CN1–3). Scale bars represent 50 μm.

We subsequently performed the RNP-based delivery of PRECISE editing on the same *ELANE*-CN patient samples as in our previous experiments. The base editing efficiency was 77%, 72%, and 68% 3 days post electroporation and reached 83%, 75%, and 84% after 14 days of neutrophilic differentiation for CN1, CN2, and CN3 patients, respectively (Figures 6A, 6B, S6A, and S6B), without detectable cytotoxicity at 24, 48, and 72 h post electroporation, compared to mock-electroporated controls (Figure 6C). PRECISE-edited cells produced a significantly elevated number of CFU-G colonies and a significantly increased percentage and absolute number of CD45^+^CD16^+^CD66b^+^ neutrophils compared to mock-electroporated controls (Figures 6D–6I).

To investigate whether the increased efficacy of ABE RNP delivery in *ELANE*-CN HSPCs represents a general phenomenon, or is specific for *ELANE*-CN patients only, we electroporated CD34^+^ HSPCs from two HDs (HD5 and HD6) with mRNA- or RNP-based PRECISE and assessed editing efficiency at 24, 48, 72, 96, and 120 h post electroporation (Figure S6C). Editing efficiency was detectable as early as 24 h post electroporation for both mRNA (HD5: 87%, HD6: 91%) and RNP formats (HD5: 76%, HD6: 72%). The editing efficiency increased steadily, reaching 91%, 95% (HD5 and HD6, respectively) in mRNA-edited cells and 82%, 81% (HD5 and HD6, respectively) in RNP-edited samples at 120 h post electroporation. Taken together, the enhanced editing efficiency observed with RNP-based ABE delivery is specific to *ELANE*-CN HSPCs.

Collectively, these results demonstrate that RNP form of PRECISE is superior to mRNA delivery in *ELANE*-CN HSPCs, exhibiting significantly higher on-target editing efficiency.

## Discussion

This study describes the development of a DSB-free ABE-mediated strategy of our previously reported gene therapy approach for *ELANE*-CN, where we successfully inhibited *ELANE* expression and restored granulocytic differentiation *in vitro* and *in vivo* through CRISPR-Cas9n double-nickase editing of the *ELANE* TATA box in patients’ HSPCs.^6^ In the current study, we further minimized the risk of genomic interference in gene-edited cells by using ABEs to convert only two adenines to guanines within the TATA box of *ELANE* using the PRECISE approach. These minor conversions were sufficient to efficiently reduce *ELANE* mRNA transcription by up to 6.6-fold and restore granulopoiesis of *ELANE*-CN HSPCs *in vitro*, without affecting integrity of HD HSPCs and their derivative neutrophils. ABEs offer a promising platform for a wide range of genetic disorders, given that G·C to A·T transitions are the most frequent pathogenic single-nucleotide polymorphisms in the ClinVar database, accounting for approximately 48% of reported variants.^10^ Several clinical trials using ABE are currently running. For example, BEAM-101, *ex vivo* base editing of HSPCs for autologous transplantation for sickle cell disease, is currently in phase 1/2 clinical trial (NCT05456880). It applies ABEs to inhibit the expression of the transcriptional repressor *BCL11A*, thereby upregulating fetal hemoglobin expression and mitigating disease pathology.^22^ Furthermore, *in vivo* single-administration of the liver-directed ABEs to inhibit *PCSK9* expression is currently being evaluated in a clinical trial phase 1b (NCT05398029) for patients with heterozygous familial hypercholesterolemia, atherosclerotic cardiovascular disease, and uncontrolled hypercholesterolemia.^23^ It is conceivable that ABE-mediated inhibition of *ELANE* expression can be developed further into clinical trial. For this, the efficacy and safety profiles should correspond to the standards appropriate for clinical application.

We observed a significant difference in the on-target efficiency of mRNA-delivered PRECISE in HSPCs from HDs compared to 6-fold lower editing in HSPCs from *ELANE*-CN patients. Base editing was variable between patients carrying different *ELANE* mutations. Simultaneously, gene editing after PRECISE RNP-based delivery reached over 68% in HSPCs, which was independent of *ELANE* mutation positions. One explanation for the discrepancy between editing efficiency of mRNA- and RNP-based ABE may involve diminished efficiency of the ABE protein translation from mRNA in *ELANE*-CN HSPCs, compared to HDs, most probably due to intracellular defects, such as protein misfolding, endoplasmic reticulum stress, or cell cycle defects, caused by inherited *ELANE* mutations. Different *ELANE* mutations may cause distinct degrees of intracellular signal transduction deregulation that result in different editing potentials. The comparable neutrophil differentiation efficiency between mRNA and RNP-based ABE, despite varying editing efficiencies at the initial stage of granulocytic differentiation *in vitro*, supports a differentiation advantage for two out of three analyzed *ELANE*-CN patients after suppression of *ELANE* expression. For the CN3 patient, we did not observe an increase in gene-edited cells after 14 days of differentiation culture. This is most probably due to the low degree of differentiation of the CN3 patient’s cells, as can be seen in cytospin slides representing cell morphology, combined with the milder neutropenia phenotype that requires a low dose of G-CSF for differentiation (G-CSF was also added to the *in vitro* culture).

An essential prerequisite of gene-editing approach to become eligible for clinical translation in the settings of *ex vivo* autologous hematopoietic stem cell editing is to preserve the pool of engraftable multipotent hematopoietic stem cells. Our previous findings showed that inhibition of *ELANE* expression by targeting *ELANE* TATA box led to engraftable gene-edited HSCs.^6^ Application of ABE to target *ELANE* TATA box should not markedly destroy hematopoietic stem cell integrity. In this study, we did not observe any differences in CFU-granulocytes/erythrocytes/macrophages/megakaryocytes (GEMM) colonies, between PRECISE and mock groups. Since CFU-GEMM colonies are derived from most immature engraftable cells within an HSPCs pool, we can conclude that this property was preserved upon PRECISE editing. However, the ultimate proof of the engraftability of PRECISE-edited HSPCs can be derived from long-term *in vivo* engraftment experiments in mice or non-human primates. It has been shown that ABE editing does not affect the long-term engraftment or multipotency of transplanted edited HSCs.^13^ Moreover, ABEs could offer a potentially less genotoxic strategy compared to other methods such as lentiviral gene therapy or classical CRISPR-Cas9 technology, as they do not depend on DSBs.^24^

It is also important to see that NE-deficient neutrophils retain their functions. Indeed, we found that the main functions of PRECISE-edited neutrophils, such as the capacity to generate ROS upon stimulation with fMLP and phagocytosis of *S. aureus* or Zymosan pHrodo green bioparticles, were preserved, which is in line with our previous findings.^2^^,^^6^ Knowing that NE is one of the main components of NET,^25^ we were concerned that inhibition of NE can compromise NET formation. We therefore investigated NETosis in PRECISE-edited neutrophils and found that inhibiting *ELANE* expression does not impair the cellular stages of NETosis in human neutrophils. To our knowledge, this is the first report highlighting the functional redundancy of NE in the complex cellular process of NETosis and needs to be investigated further.

Assessment of the off-target activity using CAST-seq and OGM revealed unaffected genomic and chromosomal integrity in PRECISE-edited HSPCs. However, we detected two off-target sites with two or three mismatches in PRECISE-edited samples at the top two *in silico*-predicted sites, chr8:130168778-130168800 and chr6:135305934-135305956, located in intronic regions of the *ASAP1* and *AHI1* genes, respectively. Our transcriptomic analysis of PRECISE-edited HSPCs confirmed that the expression levels of these genes were unchanged, and the absence of overlaps with the ENCODE database or ClinVar suggests no evidence of functional consequences. No off-target sites with four mismatches were detected, presumably because Cas9n pauses longer at sites with fewer mismatches, providing sufficient time for deaminase activity. Application of a protein-based version of the base editor, rather than mRNA, has been shown to improve its off-target profile.^13^ This reported observation was further validated in our study, as OT1 was not detected in THP-1 reporter cell lines subjected to electroporation with ABE RNP using Sanger sequencing. It is crucial to compare off-target effects of mRNA- versus RNP-based ABE targeting the *ELANE* promoter in primary HSPCs before progressing into development of a clinical trial. To further ensure the safety of PRECISE editing, genome-wide base editing off-target analysis, such as CHANGE-seq-BE,^26^ should be implemented to identify and mitigate potential unintended consequences of editing.

In summary, PRECISE editing has the potential to be further investigated and ultimately developed into a universal clinical-grade *ex vivo* or *in vivo* gene therapy approach for *ELANE*-CN patients. *In vivo* gene therapy approaches targeting mobilized CD34^+^ cells via intravenous administration of ABE-loaded nanoparticles represent a promising strategy for the development of a gene therapy platform^27^^,^^28^ for *ELANE*-CN. *In vivo* gene editing of CN patients’ CD34^+^ cells could eliminate the need for potentially toxic conditioning regimens, that are required for stem cell transplantation.^28^

## Materials and methods

### Ethical approval

The study was conducted according to the declaration of Helsinki. Informed written consent was obtained from the study participants, and the ethics committee of the University Hospital Tuebingen approved the study.

### Generation of ABE8.20-m mRNA and a source for ABE8.20-m protein

ABE8.20-m (Addgene plasmid #136300, was a gift from Nicole Gaudelli; http://n2t.net/addgene:136300; RRID: Addgene_136300) was cloned into linearized pT7-PEmax backbone for *in vitro* transcription (IVT) using NEBuilder HiFi DNA Assembly Cloning Kit (New England Biolabs, #E5520S). pT7-PEmax for IVT was a gift from David Liu (Addgene plasmid # 178113; http://n2t.net/addgene:178113; RRID: Addgene_178113). ABE8.20-m sequence was amplified by PrimeSTAR Max DNA polymerase (Takara Bio, #R045B) using pT7-ABE8.20-m as a template, a forward primer correcting the T7 promoter sequence, and a reverse primer consisting of a polyA tail. For IVT, the PCR product was first purified using QIAquick PCR Purification Kit (QIAGEN, #28106). IVT was performed using the HiScribe T7 mRNA Kit with CleanCap Reagent AG (New England Biolabs, #E2080S) and Pseudo-UTP (Tebubio, #040N-1019-1) instead of UTP, according to the manufacturer’s instructions. The mRNA was purified by LiCl precipitation following a quality (Agilent RNA 6000 Pico Kit, #5067-1513) and quantity (Qubit RNA HS Assay Kit, #Q32852) determination. The produced mRNA was aliquoted and stored at −80°C upon use.

ABE8.20-m protein, with a purity of ≥90%, as determined by SDS-PAGE under reducing conditions, was provided by GenScript as a custom order.

### Electroporation of cells and assessment of gene editing efficiency

Nucleofection of THP-1 *ELANE*-HiBiT reporter cell line and CD34^+^ HSPCs was carried out using the Amaxa nucleofection system (P3 primary kit, Lonza, #V4XP-3024) according to the manufacturer’s instructions. Each nucleofection consisted of 4 μL sgRNA (100 μM stock), combined with either 3 μg ABE8.20-m mRNA or ABE8.20-m protein. Genomic DNA was isolated using QuickExtract DNA extraction kit (Lucigen, #QE09050). PCR was performed with specific primers (FWD_Primer: 5′-GGTAAACTGAGGCAGGCGAGGC-3′; REV_Primer: 5′-AGAGGTGGGAACAGAACCCGGG-3′) and GoTaq Hot Start Polymerase (Promega, #M5006). Purification of the PCR product was conducted with the mastermix of ExoSAP, which contains 1:2 dilution of exonuclease I 20 U/mL (Thermo Fisher Scientific, #EN0581) and FastAP thermosensitive alkaline phosphatase 1 U/mL (Thermo Fisher Scientific, #EF0651). Gene editing efficiency was assessed by Sanger sequencing of the targeted *ELANE* promoter region. Sanger sequencing was performed by Microsynth, and base editing efficiency was quantified with a Python program to quantify base editing from the Sanger sequencing (BEAT) web tool.^16^

### RealTime-Glo MT cell viability assay

The RealTime-Glo MT cell viability assay (Promega, #G9711) was performed according to the manufacturer’s protocol to assess cell viability. 1,000 or 10,000 HSPCs were seeded per well in a 96-well plate (Greiner Bio-One, #655098) following electroporation and incubated at 37°C with 5% CO_2_. Luminescent signals were recorded at 24, 48, and 72 h post electroporation using a GloMax multi-detection plate reader with a 0.5-s integration time.

### Endogenous HiBiT-tagged NE protein quantification

Endogenous HiBiT-tagged NE protein was measured in a white 96-well tissue culture plate (Greiner Bio-One, #655075) according to the manufacturer’s instructions. Briefly, 1 × 10^4^ cells in 50 μL complete medium (RPMI supplemented with 10% FCS, 1% penicillin/streptomycin) were lysed with 50 μL Nano-Glo HiBiT lytic reagent (Promega, #N3030) for 30 min at room temperature (RT) while shaking. Luminescence signal was quantified using a GloMax multidetection system plate reader with a 0.5-s integration time.

### Western blotting

A total of 1 × 10^6^ cells were lysed in 200 μL 3× Laemmli buffer, and protein was denatured for 10 min at 95°C. 20 μL of cell lysates were loaded on 15% polyacrylamide gel per lane. Proteins were transferred on a nitrocellulose membrane (GE Healthcare, #10600002) for 1 h at 100 V, 4°C. Blocking of the membrane was performed for 1 h in 5% blocking milk/TBST and membrane was incubated overnight at 4°C with primary anti-NE (Abcam, #ab131260) or α-Tubulin (Cell Signaling, #2144s) antibody. Subsequently, the membranes were washed and incubated with the secondary horseradish peroxidase-conjugated antibody (Cell Signaling, #7074s) for 1 h at RT. The chemiluminescence signal was measured with VILBER Imaging system.

### Isolation and expansion of primary human CD34^+^ HSPCs

Bone marrow samples from three *ELANE*-CN patients were collected during the routine annual follow-up recommendation by the Severe Chronic Neutropenia International Registry (SCNIR,) and cord blood samples from four HDs were obtained from University Women’s Hospital Tübingen. The study was conducted according to the Declaration of Helsinki. Informed written consent was obtained from study participants, and the ethics committee of the University Hospital Tübingen approved the study. CD34^+^ HSPCs were isolated from the bone marrow mononuclear cell fraction of *ELANE*-CN patients and cord blood of HDs by Ficoll gradient centrifugation and magnetic bead separation using the CD34 MicroBead Kit (Miltenyi Biotech, #130-046-703). CD34^+^ cells were expanded for up to 7 days in CD34^+^ cell expansion medium (StemSpan SFEM II, STEMCELL Technologies, #09655) supplemented with 1% penicillin/streptomycin, 1% glutamine, and cytokines: 20 ng/mL IL-3 (BioLegend, #578006), 20 ng/mL IL-6 (R&D Systems, #7270-IL-100/CF), 20 ng/mL TPO (BioLegend, #763706), 50 ng/mL SCF (R&D Systems, #11010-SC-100), and 50 ng/mL FLT3L (BioLegend, #550606).

### Liquid culture granulocytic differentiation of primary human CD34^+^ HSPCs

CD34^+^ HSPCs from *ELANE*-CN patients or HDs were cultured for 7 days in differentiation medium 1 (RPMI supplemented with 10% FCS, 1% penicillin/streptomycin, and human recombinant cytokines: 5 ng/mL SCF [R&D Systems, #11010-SC-100], 5 ng/mL IL-3 [BioLegend, #578006], 5 ng/mL GM-CSF [PeproTech, #300-03], and 1 ng/mL G-CSF [Lenograstim, Granocyte, Chugai Pharma, PZN 7253193]) at a density of >2 × 10^5^ cells/mL, followed by 7 days in differentiation medium 2 (RPMI supplemented with 10% FCS, 1% penicillin/streptomycin, and 1 ng/mL G-CSF [Lenograstim, Granocyte, Chugai Pharma, PZN 7253193]). Half of the differentiation medium was refreshed every second day for the entire liquid culture differentiation (LCD) procedure. After 14 days, 5 × 10^4^ cells were used for morphological analysis, and 1.5 × 10^5^ cells were used for flow cytometry analysis after staining of cells with the following mouse anti-human antibodies: CD45-BV510 (BioLegend, #304036), CD34-PE-Cy7 (BD, #348811), CD33-BV421 (BioLegend, 303416), CD15-PE (BioLegend, 323006), CD16-APC (BioLegend, #302012), CD66b-FITC (BioLegend, #305104), CD11b-APC-Cy7 (BD, #557754), and 7AAD (BD, #559925). Anti-mouse IgGk beads (BD, #51-90-9001229) were used for compensation. Samples were analyzed using FACSCanto II (BD) and FlowJo software v.10 (BD). For morphological analysis, 50,000 suspension cells from day 14 of LCD were centrifuged in cytospin centrifuge for 4 min at 250 rpm onto glass slides and stained with May-Grunwald solution (Sigma, #1.01424.1000) for 5 min and Giemsa solution (Sigma, #1.09204.1000) for 20 min.

### CFU assay

1,500 CD34^+^ HSPCs were resuspended in 300 μL Iscove’s modified Dulbecco’s medium supplemented with 2% FBS (STEMCELL Technologies, #07700) and added to 3 mL methocult H4435 enriched medium (STEMCELL Technologies, #04435). 1.1 mL of cell suspension was plated per 35 mm cell culture dish (Nunc, #150318). Dishes were cultured next to a PBS-loaded dish for 14 days at 37°C and 5% CO_2_. Colonies were counted after 12–14 days.

### ROS assay

Granulocytes harvested on day 14 of LCD were seeded at a density of 5 × 10^4^ cells per well in a 96-well white-walled plate in 80 μL RPMI 1640 medium supplemented with 0.5% BSA. Cells were stimulated with 1 μM fMLP (Sigma, #F3506) or left untreated for 30 min at 37°C, 5% CO_2_. Levels of hydrogen peroxide (H_2_O_2_), a form of Reactive Oxygen Species (ROS), were measured using the ROS-Glo H_2_O_2_ assay kit (Promega, #G8820) on a GloMax multidetection system plate reader, following the manufacturer’s protocol.

### Phagocytosis assay

Granulocytes harvested on day 14 of LCD were seeded at a density of 1 × 10^4^ cells per well in a 96-well plate (Corning, #3596) in 90 μL of RPMI 1640 medium supplemented with 0.5% BSA. 10 μg of pHrodo Green *S. aureus* bioparticles (Essen Bio, #4620) or 5 μg pHrodo Zymosan bioparticles (Essen Bio, #4618) were added to a final volume of 100 μL. Cells were incubated at 37°C, 5% CO_2_ in an IncuCyte ZOOM system (Essen BioScience) for 12 h. Phagocytosis was monitored and analyzed using IncuCyte S3 Software, following the manufacturer’s protocol.

### *In vitro* NET formation assay

3 × 10^4^ *in vitro*-generated neutrophils were resuspended per well in HEPES (10 mM; Gibco #15630-080) and BSA (0.05%; Roth, #1ET6.3) supplemented phenol red-free RPMI 1640 medium (Gibco, #11835-063) and plated on a coverslip (Roth, #YX03.2) in a 24-well plate (Corning, #3524). After allowing the cells to adhere for 30 min at 37°C and 5% CO_2_, the cells were left unstimulated or stimulated with ionomycin (4 μM; Invitrogen, #I24222) or PMA (100 nM; Merck, #524400) for 4 h at 37°C and 5% CO_2_. Following activation, fixation was performed in 1.3% PFA at RT for 30 min and cells were subsequently washed twice with PBS, permeabilized (0.1% Triton X-100, 0.1% BSA in 1× PBS) for 10 min at 4°C, and incubated with blocking buffer (2.5% BSA, 0.5% Tween 20 in 1× PBS) at 37°C for 1 h. Afterward, samples were incubated at 4°C overnight with the primary antibodies against H3cit (1:1,000; Abcam, #ab219407) and NE (1:1,000; DAKO, #M0752), washed 2 times the following day with PBS before incubation with the corresponding secondary antibodies (1:1,500; Invitrogen, #A21428 and #A31574) for 2 h at RT. After another 2 washing steps with PBS, the coverslips were mounted using mounting medium containing DAPI (Invitrogen, #P36962) and visualized on a Nikon Eclipse Ti2 microscope. The percentage of NET and morphological changes were analyzed from 5 non-overlapping and randomized visual fields per well. NETosis was analyzed by quantifying cells with a web-like chromatin structure and positive citrullinated histone H3 staining.

### rhAmpSeq

Targeted amplicon sequencing was performed using the IDT rhAmpSeq CRISPR analysis system. Briefly, rhAmp primers targeting potential off-target sites (Table 1) were designed using IDT’s rhAmp primer design software and ordered from IDT. Library preparation was performed according to the manufacturer’s protocol, followed by next-generation sequencing (NGS) at Novogene. The CRISPRESSO package^18^ was used to analyze the data. All genomic coordinates are based on the human reference genome assembly GRCh38.

### CAST-seq

Genomic DNA was isolated 5 days post electroporation of HD HSPCs edited with mock or PRECISE using the QIAamp DNA Micro Kit (QIAGEN, #56304). CAST-seq library preparation for NGS and data analysis were performed as described by Turchiano et al.,^29^ with an improved bioinformatics pipeline.^30^ Three biological replicates were analyzed, and OMTs presented in at least two of three biological replicates were reported.

### OGM

Ultra-high molecular weight (UHMW) gDNA was extracted according to the manufacturer’s guidelines (Bionano Genomics, San Diego, CA, USA, and Bionano Prep Cell Culture DNA Isolation ProtocolCG-00006 Rev B, Bionano Genomics, San Diego, CA, USA) using 1.5 × 10^6^ HSPCs 5 days post electroporation with mock or PRECISE. UHMW gDNA was quantified using the Qubit dsDNA BR Assay Kit and a Qubit 3.0 Fluorometer (Thermo Fisher Scientific, Waltham, MA, USA). To ensure the homogeneity of the gDNA, the concentration was determined at three different points in the tube (left, middle, and right side). Isolated gDNA with a concentration equal or above 40 ng/μL and a coefficient of variation < 0.3 was then labeled according to the manufacturer’s guidelines (Bionano Prep Direct Label and Stain Protocol CG-30553-1 Rev E). Purified UHMW gDNA (750 ng) was first labeled with DL-green fluorophores using the DLE-1 enzyme. Proteinase K digestion (Bionano Genomics, San Diego, CA, USA) was performed, and DLE was cleaned up using a membrane on a microplate. The backbone of the DNA was labeled using DTT and DNA stain (DNA stain reagent, Bionano Genomics, San Diego, CA, USA). Then, the sample was rotated on the HulaMixer and stored protected from light until quantification. The labeled gDNA with a required concentration of 4–16 ng/μL was loaded on the Saphyr G3.3 chips, and molecules were imaged by the Saphyr instrument with a minimal capacity of 1,000 GB per sample.

The Bionano Access software was used for the *de novo* assemblies and structural variant calling (Tools version Solve3.8.2, Bionano Genomics, San Diego, CA, USA). After the molecule files of each sample were generated, the data were filtered down to 500 GB with a minimal molecule length of 150 kb. From this filtered file, a *de novo* assembly was done for each sample. The data were further filtered with the following confidence filter settings: the confidence levels for insertions, deletions, and duplications were set to 0.7. The frequency of SVs present in the Bionano control sample cohort was set to 1%. The self-molecule count (minimal amount of molecules supporting the variant) was set to 5. As reference genome hg38 was used. To identify subclonal changes, the Rare Variant Pipeline (Tools version Solve3.8.2, Bionano Genomics, San Diego, CA, USA) with the total throughput of each run was used. In the dual analysis, HD2 PRECISE sample was measured against HD2 mock to call variants that were detected in the gene-edited sample but not in the mock.

### Statistical analysis

For cell experiments, differences in mean values between two groups were analyzed using two-sided, unpaired Student’s *t* test with GraphPad Prism 9 or Microsoft Excel 2019 software. One-way ANOVA with Dunnett’s or Tukey’s test was applied for multiple group comparison with one variable. Two-way ANOVA with Tukey’s test was used for multiple group comparisons with two variables comparing all means with each other.

## Data availability

For original data, please contact julia.skokowa@med.uni-tuebingen.de.

## Acknowledgments

This work was supported by the 10.13039/501100002347German Federal Ministry of Education and Research (BMBF), the Madeleine Schickedanz-Kinderkrebsstiftung, the 10.13039/501100005972German Cancer Aid, the 10.13039/501100001659German Research Foundation (DFG: CRC1597 – project A05), the German Federal Ministry of Research, Technology and Space (BMFTR), the SPARK-BIH program, and the 10.13039/100000002National Institutes of Health. We thank the Flow Cytometry Core Facility of the University Hospital Tübingen for technical support and Geoffroy Andrieux (Medical Center – University of Freiburg) for bioinformatic support.

## Author contributions

M.N., B.F., and J.S. made initial observations and designed the experiments; J.S. acquired funding, provided resources, and supervised the project. B.D., M.N., and B.F. performed granulocytic differentiation and CFU assays of primary CN and HD HSPCs and analyzed data; M.N., F.B., and B.F. performed phagocytosis and ROS assay and analyzed the data. F.M., P.M., and O.B. performed NET formation assay and analyzed the data. M.N. and B.F. performed rhAmpSeq *in silico* analysis of predicted off-target sites and analyzed the data; M.N., M.K., and S.K. performed the RNA-seq and analyzed the data. M.M.K., S.A., and T.C. performed CAST-seq and analyzed the data; I.K. and D.S. performed OGM and analyzed the data; K.W. and C.Z. provided patients’ material; M.N., B.D., B.F., and J.S. interpreted the results and wrote the manuscript. All authors reviewed and revised the manuscript. K.W., C.Z., C.L., and T.C. gave insightful comments.

## Declaration of interests

M.N., B.F., B.D., and J.S. have filed a patent on the invention described in this study. T.C. holds a patent on CAST-seq.

## Supplemental information

Document S1. Figures S1–S7

Document S2. Article plus supplemental information
