# Supplementary material for: Targeted inhibition of ELANE expression using adenine base editing to treat severe congenital neutropenia
Source: Mol Ther Methods Clin Dev. 2025 Nov 5;33(4):101626. doi: 10.1016/j.omtm.2025.101626 (PMC12719991; doi:10.1016/j.omtm.2025.101626)
Supplement: Document S1. Figures S1–S7 [file mmc1.pdf]

OMTM, Volume *33*

Supplemental information

Targeted inhibition of *ELANE* expression using adenine base editing to treat severe congenital neutropenia

Betül Findik, Benjamin Dannenmann, Franka Bernhard, Masako Monika Kaufmann, Sandra Ammann, Sergey Kandabarau, Maksim Klimiankou, Fabian Mauch, Patrick Münzer, Oliver Borst, Isabel Klefenz, Doris Steinemann, Claudia Lengerke, Cornelia Zeidler, Toni Cathomen, Karl Welte, Masoud Nasri, and Julia Skokowa

A

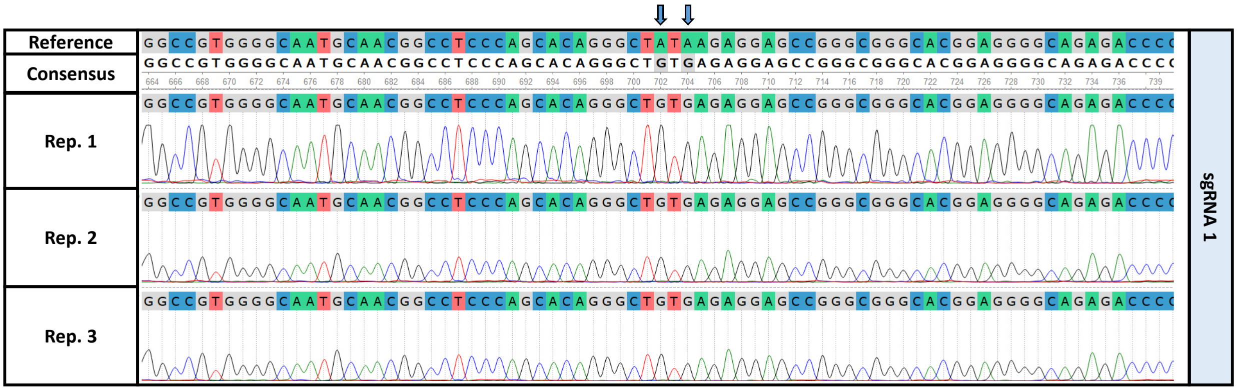

B

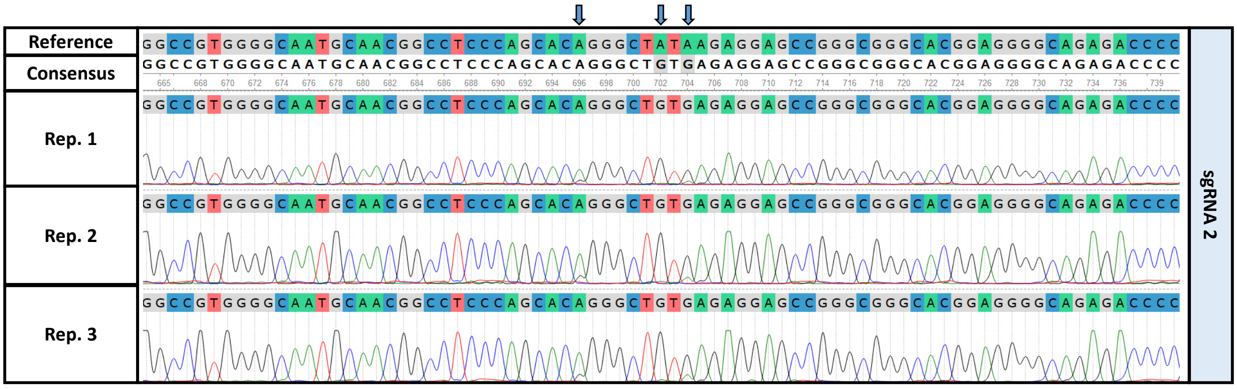

C

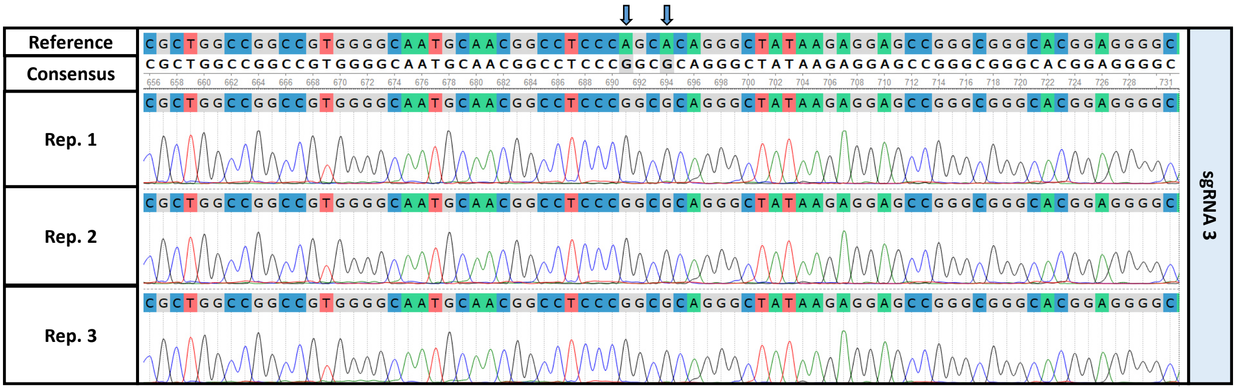

D

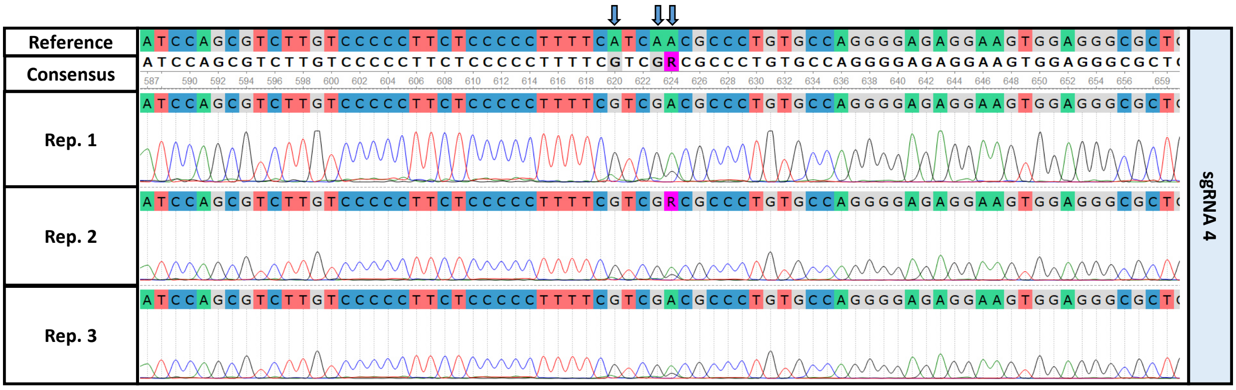

Figure S1

Figure S1. Representative Sanger sequencing traces of base-editing with *ELANE* promoter targeting sgRNAs (related to Figure 1)

A-D. Representative Sanger sequencing traces of DNA isolated from NE-HiBiT-tagged THP-1 reporter cell lines five days post-electroporation with ABE8.20-m mRNA and *ELANE* promoter targeting sgRNAs. Arrows indicate the target sites for base editing within the DNA sequence. Replicates (Rep) 1 to 3 represent data from three independent experiments for the respective sgRNA. Alignment was performed using Unipro UGENE: a unified bioinformatics toolkit.

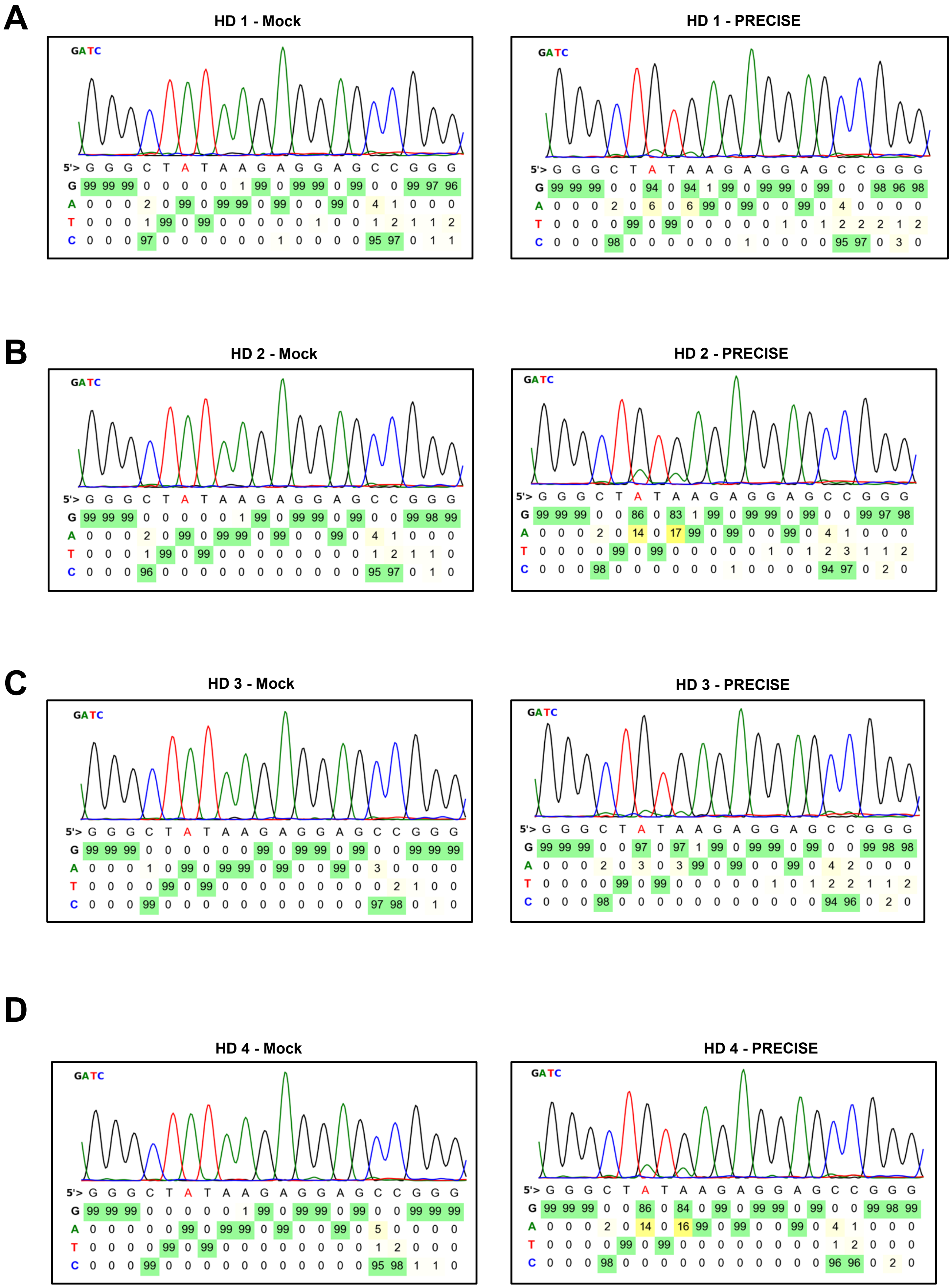
A
HD 1 - Mock
GATC
5'> G G G C T A T A A G A G G A G C C G G G
G 99 99 99 0 0 0 0 0 1 99 0 99 99 0 99 0 0 99 97 96
A 0 0 0 2 0 99 0 99 99 0 99 0 0 99 0 4 1 0 0 0
T 0 0 0 1 99 0 99 0 0 0 0 0 1 0 0 1 2 1 1 2
C 0 0 0 97 0 0 0 0 0 0 1 0 0 0 0 95 97 0 1 1
HD 1 - PRECISE
GATC
5'> G G G C T A T A A G A G G A G C C G G G
G 99 99 99 0 0 94 0 94 1 99 0 99 99 0 99 0 0 98 96 98
A 0 0 0 2 0 6 0 6 99 0 99 0 0 99 0 4 0 0 0 0
T 0 0 0 0 99 0 99 0 0 0 0 0 1 0 1 2 2 2 1 2
C 0 0 0 98 0 0 0 0 0 0 1 0 0 0 0 95 97 0 3 0
B
HD 2 - Mock
GATC
5'> G G G C T A T A A G A G G A G C C G G G
G 99 99 99 0 0 0 0 0 1 99 0 99 99 0 99 0 0 99 98 99
A 0 0 0 2 0 99 0 99 99 0 99 0 0 99 0 4 1 0 0 0
T 0 0 0 1 99 0 99 0 0 0 0 0 0 0 0 1 2 1 1 0
C 0 0 0 96 0 0 0 0 0 0 0 0 0 0 0 95 97 0 1 0
HD 2 - PRECISE
GATC
5'> G G G C T A T A A G A G G A G C C G G G
G 99 99 99 0 0 86 0 83 1 99 0 99 99 0 99 0 0 99 97 98
A 0 0 0 2 0 14 0 17 99 0 99 0 0 99 0 4 1 0 0 0
T 0 0 0 0 99 0 99 0 0 0 0 0 1 0 1 2 3 1 1 2
C 0 0 0 98 0 0 0 0 0 0 1 0 0 0 0 94 97 0 2 0
C
HD 3 - Mock
GATC
5'> G G G C T A T A A G A G G A G C C G G G
G 99 99 99 0 0 0 0 0 0 99 0 99 99 0 99 0 0 99 99 99
A 0 0 0 1 0 99 0 99 99 0 99 0 0 99 0 3 0 0 0 0
T 0 0 0 0 99 0 99 0 0 0 0 0 0 0 0 0 2 1 0 0
C 0 0 0 99 0 0 0 0 0 0 0 0 0 0 0 97 98 0 1 0
HD 3 - PRECISE
GATC
5'> G G G C T A T A A G A G G A G C C G G G
G 99 99 99 0 0 97 0 97 1 99 0 99 99 0 99 0 0 99 98 98
A 0 0 0 2 0 3 0 3 99 0 99 0 0 99 0 4 2 0 0 0
T 0 0 0 0 99 0 99 0 0 0 0 0 1 0 1 2 2 1 1 2
C 0 0 0 98 0 0 0 0 0 0 0 0 0 0 0 94 96 0 2 0
D
HD 4 - Mock
GATC
5'> G G G C T A T A A G A G G A G C C G G G
G 99 99 99 0 0 0 0 0 1 99 0 99 99 0 99 0 0 99 99 99
A 0 0 0 0 0 99 0 99 99 0 99 0 0 99 0 5 0 0 0 0
T 0 0 0 0 99 0 99 0 0 0 0 0 0 0 0 1 2 0 0 0
C 0 0 0 99 0 0 0 0 0 0 0 0 0 0 0 95 98 1 1 0
HD 4 - PRECISE
GATC
5'> G G G C T A T A A G A G G A G C C G G G
G 99 99 99 0 0 86 0 84 0 99 0 99 99 0 99 0 0 99 98 99
A 0 0 0 2 0 14 0 16 99 0 99 0 0 99 0 4 1 0 0 0
T 0 0 0 0 99 0 99 0 0 0 0 0 0 0 0 1 2 0 0 0
C 0 0 0 98 0 0 0 0 0 0 0 0 0 0 0 96 96 0 2 0

Figure S2

Figure S2. Base-editing analysis tool (BEAT) output for mock and PRECISE-edited healthy donor-derived CD34$^+$ cells (related to Figure 2)

A-D. Original BEAT analysis result for on-target activity of PRECISE. Sanger sequencing traces of DNA isolated from mock and PRECISE (mRNA format) five days post-electroporation from four biological replicates (HD 1-4) used for the analysis. The calculated percentage of each base at each position is depicted.

A

OT1- chr8:130168778-130168800

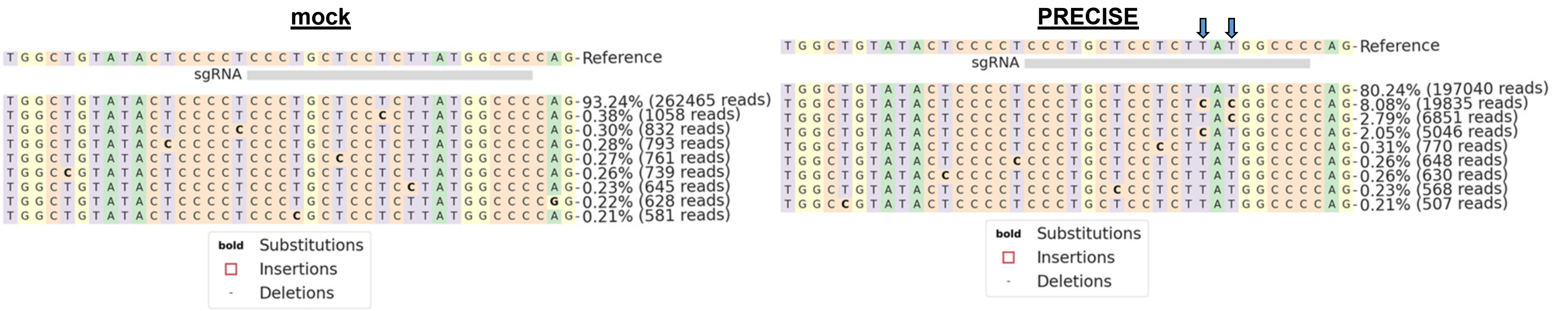

B

OT2- chr6:135305934-135305956

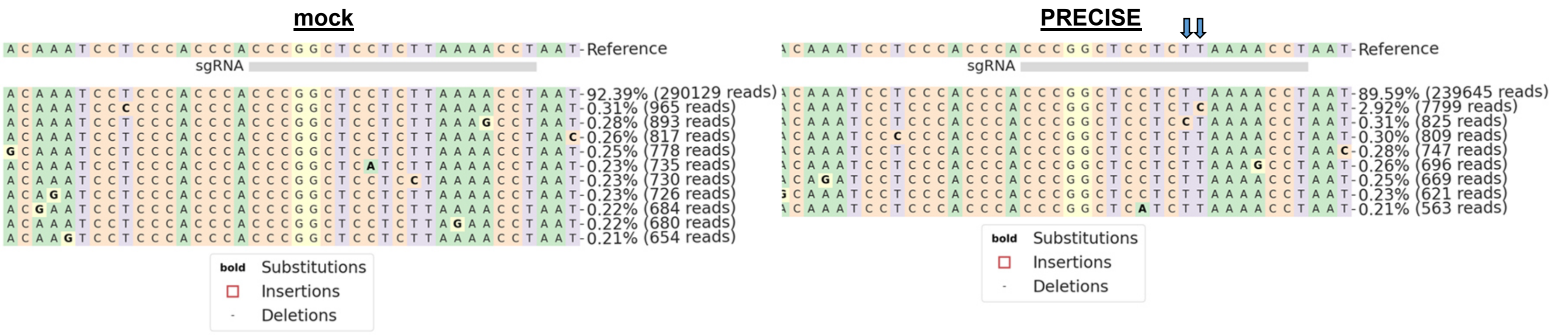

C

Category	Mock	PRECISE	Dual analysis
Insertion	30	24	0
Deletion	50	34	0
Inversion	4	0	0
Duplication	0	0	0
Intra-Fusion	0	0	0
Inter-Translocation	0	0	0
AOH/LOH Region	0	1	1
CNV Gain Segment	0	1	1
CNV Loss Segment	0	0	0
Aneuploidy Gain	0	0	0
Aneuploidy Loss	0	0	0

Figure S3

Figure S3. Assessment of PRECISE off-target profile in human primary HSPCs by rhAmpSeq and Optical genome mapping (related to Figure 4)

A-B. PRECISE off-target activity at OT1 and 2 in healthy donor's primary HSPCs (HD1-3). Deep-seq sequencing data generated from mock or PRECISE (mRNA form) treated HDs five days post-electroporation, as assessed using the CRISPResso package. Sequencing data from HD1-3 (n=3) were pooled before analysis. Alignment and editing frequency of reads, determined by the percentage and number of the sequence reads. Arrows indicate the off-target activity sites. **C.** List of different categories of SVs and their numbers detected by OGM in Mock, PRECISE and dual analysis (PRECISE versus Mock).

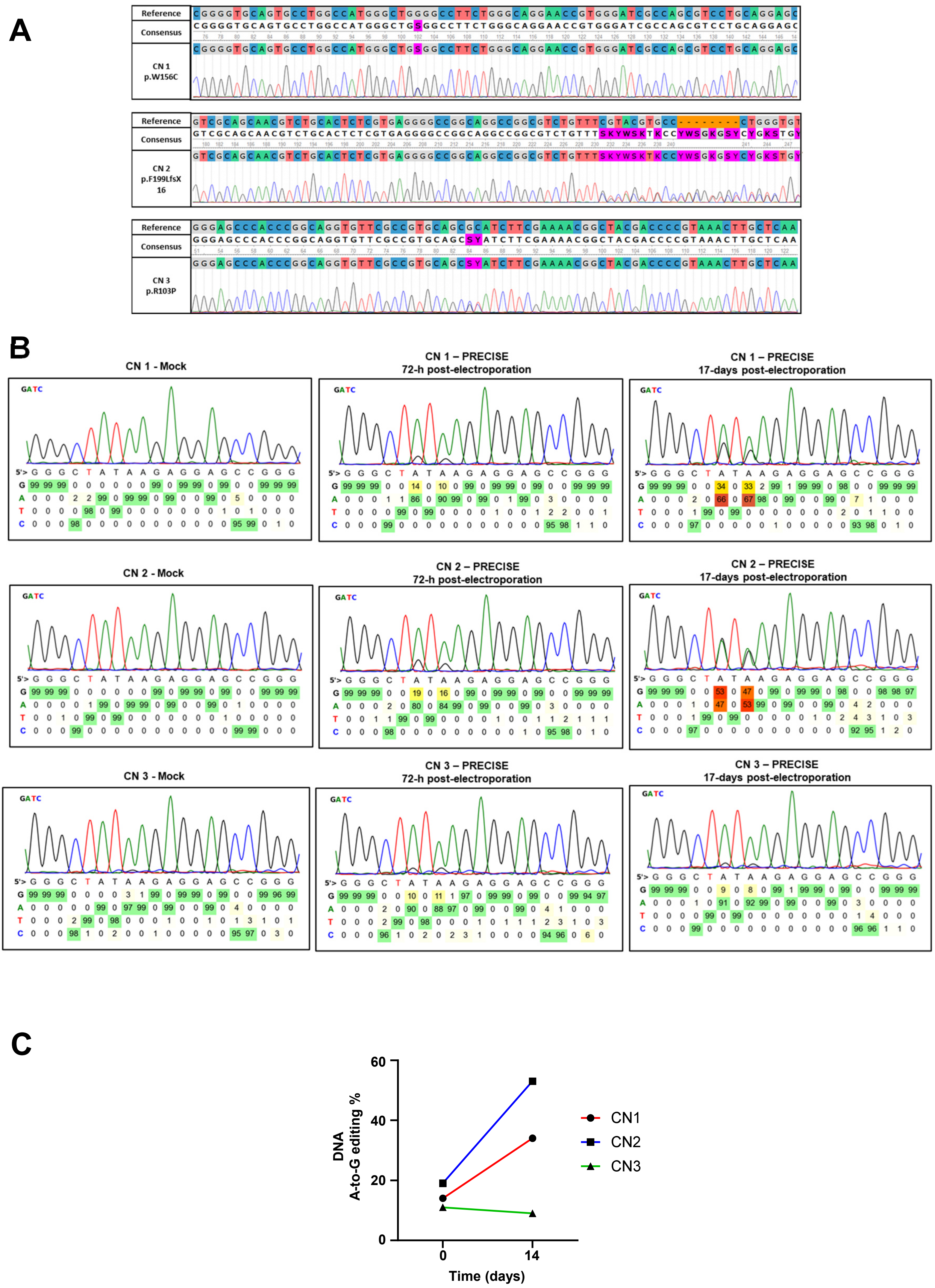
A
Reference
Consensus
CN 1
p.W156C
CN 2
p.F199LfsX
16
CN 3
p.R103P
B
CN 1 - Mock
CN 1 – PRECISE
72-h post-electroporation
CN 1 – PRECISE
17-days post-electroporation
CN 2 - Mock
CN 2 – PRECISE
72-h post-electroporation
CN 2 – PRECISE
17-days post-electroporation
CN 3 - Mock
CN 3 – PRECISE
72-h post-electroporation
CN 3 – PRECISE
17-days post-electroporation
GATC
5'> G G G C T A T A A G A G G A G C C G G G
C
DNA
A-to-G editing %
Time (days)
60
40
20
0
0
14
CN1
CN2
CN3

Figure S4

Figure S4. CD34$^+$ *ELANE*-CN HSPCs mutation and Base-editing analysis tool (BEAT) output for mock and PRECISE (mRNA form) (related to Figure 5)

A. Representative Sanger sequencing chromatograms showing mutations in the *ELANE* gene for CN patients (CN1-3). Alignment was performed using Unipro UGENE: a unified bioinformatics toolkit. **B**. BEAT analysis of on-target activity of PRECISE. Sanger sequencing traces of DNA isolated from mock and PRECISE (mRNA format) three- and seventeen-days post-electroporation from three biological replicates (CN 1-3) used for the analysis. The calculated percentage of each base at each position is depicted. **C.** Gene editing results showing on-target activity of PRECISE (mRNA format) during granulocytic differentiation from three biological replicates (CN 1-3). The graph illustrates the percentage of on-target activity at Day 0 (start) and Day 14 (end) of the differentiation protocol. Due to bystander editing event, the highest calculated percentage of each sample is depicted. Red, blue, and green lines correspond to CN 1, CN 2, and CN 3, respectively.

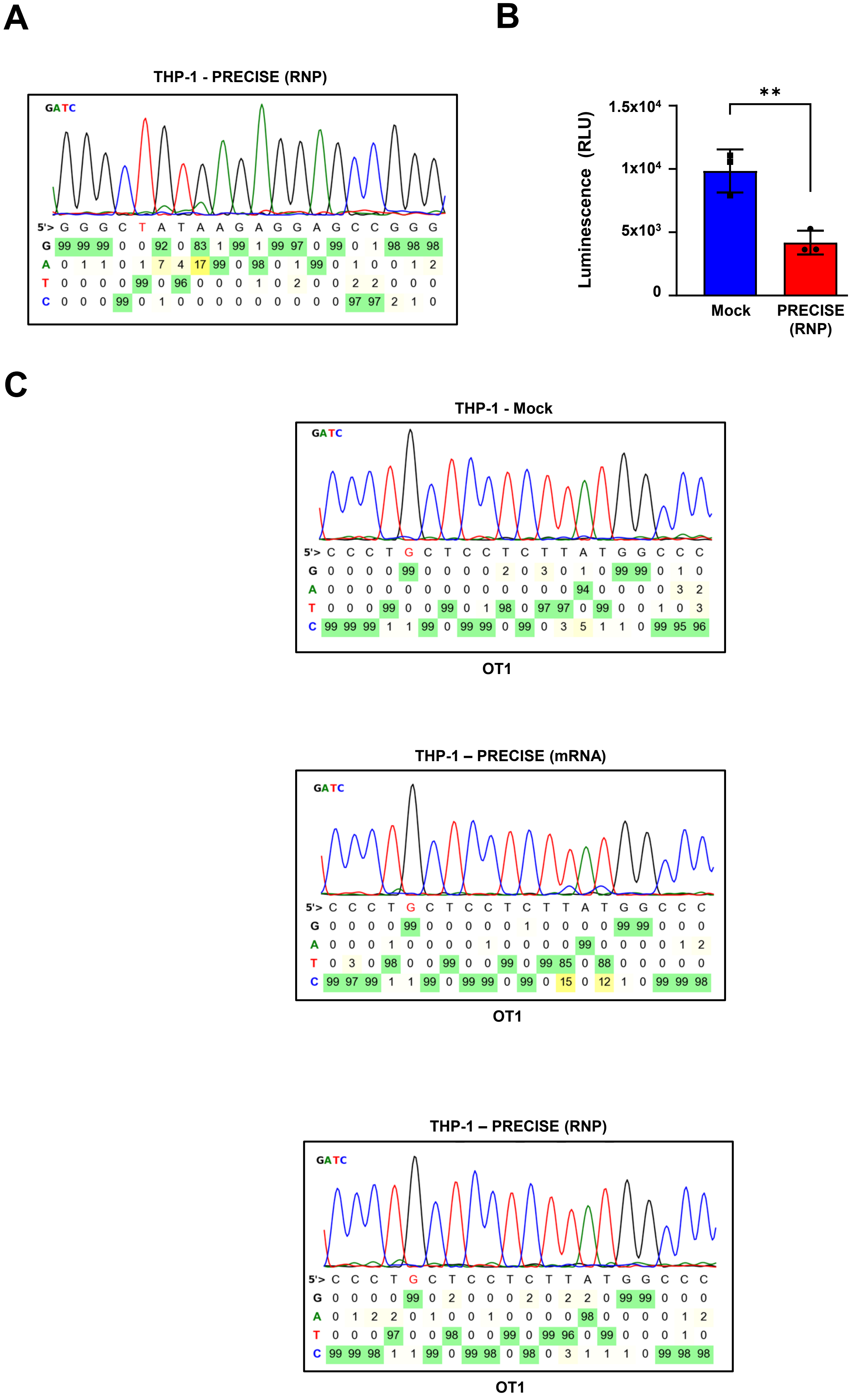
A
THP-1 - PRECISE (RNP)
GATC
5'> G G G C T A T A A G A G G A G C C G G G
G 99 99 99 0 0 92 0 83 1 99 1 99 97 0 99 0 1 98 98 98
A 0 1 1 0 1 7 4 17 99 0 98 0 1 99 0 1 0 0 1 2
T 0 0 0 0 99 0 96 0 0 0 1 0 2 0 0 2 2 0 0 0
C 0 0 0 99 0 1 0 0 0 0 0 0 0 0 0 97 97 2 1 0
B
Luminescence (RLU)
1.5x10^4
1x10^4
5x10^3
0
**
Mock
PRECISE (RNP)
C
THP-1 - Mock
GATC
5'> C C C T G C T C C T C T T A T G G C C C
G 0 0 0 0 99 0 0 0 0 2 0 3 0 1 0 99 99 0 1 0
A 0 0 0 0 0 0 0 0 0 0 0 0 0 94 0 0 0 0 3 2
T 0 0 0 99 0 0 99 0 1 98 0 97 97 0 99 0 0 1 0 3
C 99 99 99 1 1 99 0 99 99 0 99 0 3 5 1 1 0 99 95 96
OT1
THP-1 – PRECISE (mRNA)
GATC
5'> C C C T G C T C C T C T T A T G G C C C
G 0 0 0 0 99 0 0 0 0 0 1 0 0 0 0 99 99 0 0 0
A 0 0 0 1 0 0 0 0 1 0 0 0 0 99 0 0 0 0 1 2
T 0 3 0 98 0 0 99 0 0 99 0 99 85 0 88 0 0 0 0 0
C 99 97 99 1 1 99 0 99 99 0 99 0 15 0 12 1 0 99 99 98
OT1
THP-1 – PRECISE (RNP)
GATC
5'> C C C T G C T C C T C T T A T G G C C C
G 0 0 0 0 99 0 2 0 0 0 2 0 2 2 0 99 99 0 0 0
A 0 1 2 2 0 1 0 0 1 0 0 0 0 98 0 0 0 0 1 2
T 0 0 0 97 0 0 98 0 0 99 0 99 96 0 99 0 0 0 1 0
C 99 99 98 1 1 99 0 99 98 0 98 0 3 1 1 1 0 99 98 98
OT1

Figure S5

Figure S5. RNP outperforms mRNA in on- and off-target base-editing activity

A,B. Representative BEAT analysis of on-target activity of PRECISE using RNP. Sanger sequencing traces of DNA (**A**) and NE levels (**B**) from PRECISE (RNP form) edited NE-HiBiT-tagged THP-1 reporter cell line three days post-electroporation. Luminescence signal (RLU) was quantified using the GloMax system. Data are presented as mean ± standard deviation (SD) from three independent experiments. Unpaired Student's t-test was applied for group comparison, $^{**}p < 0.01$. **C.** BEAT analysis of off-target activity at OT1 of PRECISE using mRNA or RNP. Sanger sequencing traces of DNA isolated from mock and PRECISE (mRNA or RNP form) treated NE-HiBiT-tagged THP-1 reporter cell line three days post-electroporation. The calculated percentage of each base at each position is depicted.

A

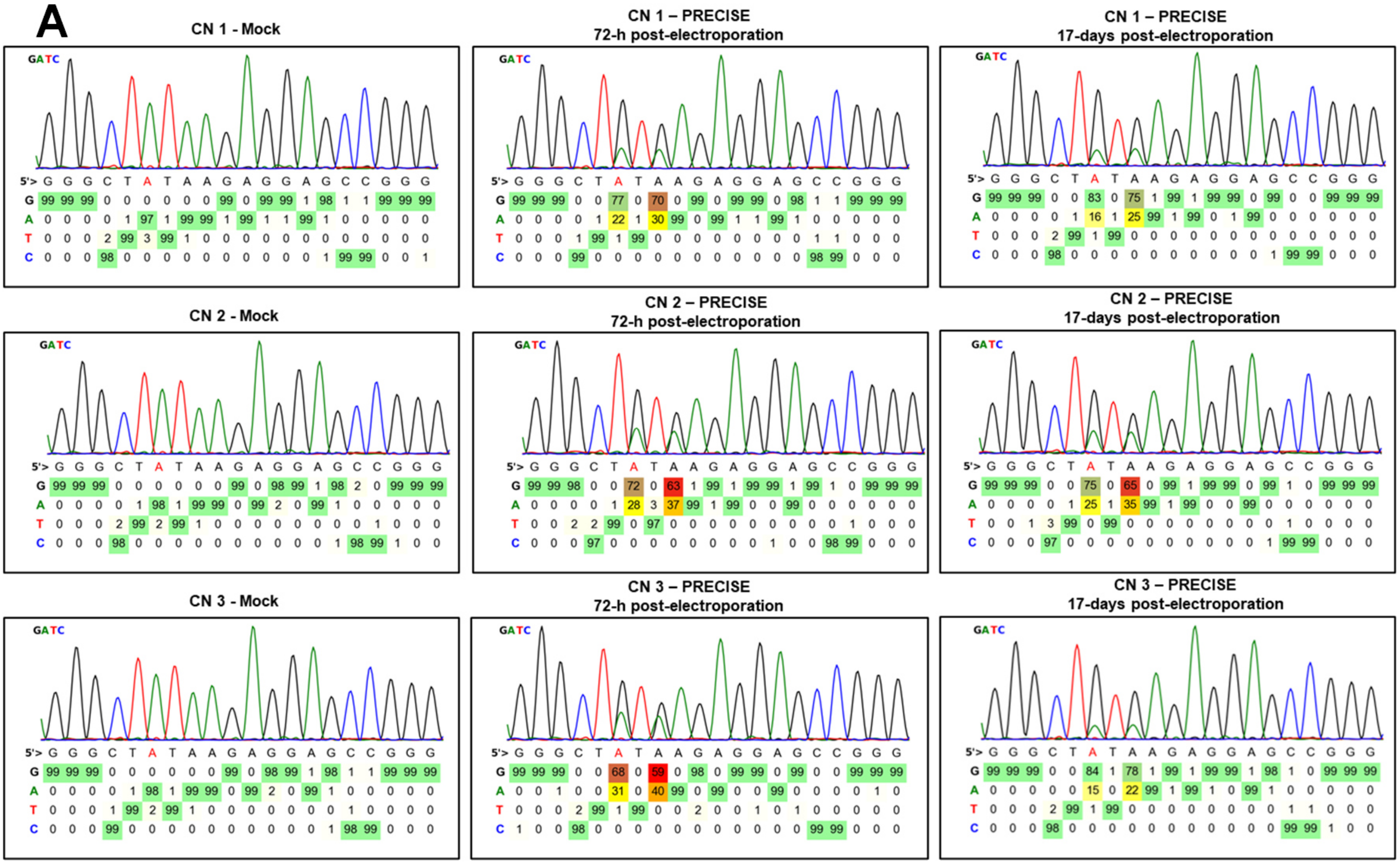

B

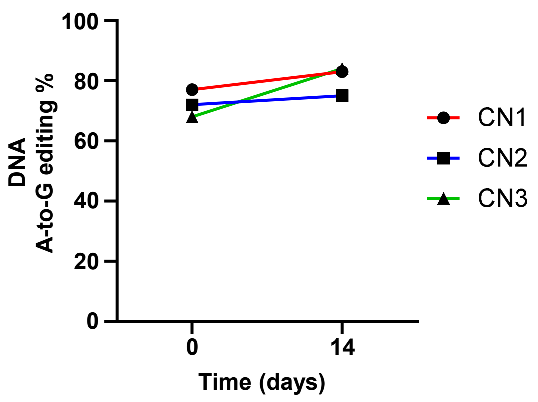

C

Time (h) / Sample	ABE8.20-m mRNA A-to-G editing %										ABE8.20-m RNP A-to-G editing %									
	THP-1	HD1	HD2	HD3	HD4	HD5	HD6	CN1	CN2	CN3	THP-1	HD1	HD2	HD3	HD4	HD5	HD6	CN1	CN2	CN3
**0**	-	-	-	-	-	0	0	-	-	-	-	-	-	-	-	0	0	-	-	-
**24**	-	-	-	-	-	87	91	-	-	-	-	-	-	-	-	76	72	-	-	-
**48**	-	-	-	-	-	92	94	-	-	-	-	-	-	-	-	83	78	-	-	-
**72**	92	-	-	-	-	91	95	14	19	10	92	-	-	-	-	84	79	77	72	68
**96**	-	-	-	-	-	90	94	-	-	-	-	-	-	-	-	84	78	-	-	-
**120**	99	94	86	97	86	91	95	-	-	-	-	-	-	-	-	82	81	-	-	-

Figure S6

Figure S6. Base-editing analysis tool (BEAT) output for mock and PRECISE (RNP form) CD34$^+$ *ELANE*-CN HSPCs (related to Figure 6)

A. BEAT analysis of on-target activity of PRECISE using RNP. Sanger sequencing traces of DNA isolated from mock and PRECISE (RNP form) three- and seventeen-days post-electroporation from three biological replicates (CN 1-3) used for the analysis. The calculated percentage of each base at each position is depicted. **B.** Gene editing results showing on-target activity of PRECISE (RNP format) during granulocytic differentiation from three biological replicates (CN 1-3). The graph illustrates the percentage of on-target activity at Day 0 (start) and Day 14 (end) of the differentiation protocol. Due to bystander editing event, the highest calculated percentage of each sample is depicted. Red, blue, and green lines correspond to CN 1, CN 2, and CN 3, respectively. **C.** Comparison of A to G on-target editing between mRNA and RNP formats of the PRECISE approach in the THP-1 *ELANE*-HiBiT reporter cell line, HD, and CN HSPCs. For samples HD 5 and HD 6, the percentage of on-target activity is shown at 24, 48-, 72-, 96-, and 120-hours post-electroporation. Due to bystander editing events, the highest calculated percentage of each sample is depicted.

A

gene	log2Fold	padj
PDE5A	1.157668996	0.020580803
VWF	1.008961975	0.014858578
FAM156A	-1.157966558	6.69E-07
ELANE	-2.723521295	1.85E-49

B

gene	log2Fold	padj
ELANE	-2.723521295	1.85E-49
AZU1	-0.16557883	0.999957957
PRTN3	0.072209308	0.854748133
CFD	0.200209625	0.999957957
MED16	0.105255922	0.999957957

C

gene	log2Fold	padj
ASAP1	0.075458751	0.999957957
AHI1	0.198397386	0.999957957

Figure S7

Figure S7. Selected genes from DESeq2 analysis of RNA-seq data in PRECISE-edited healthy donor HSPCs (related to Figure 4)

A-C. DESeq2 analysis of RNA-seq data of PRECISE-edited healthy donor HSPCs (n=3) at 5 days post-electroporation, compared to mock-electroporated controls. **(A)** Differentially expressed genes; (**B**) Expression levels of genes neighboring the *ELANE* gene; **(C)** expression levels of OT1 (the *ASAP1* gene) and OT2 (the *AHI1* gene).
